# Developing deprotectase biocatalysts for synthesis†

**DOI:** 10.1039/d4fd00016a

**Published:** 2024-03-01

**Authors:** Lisa Kennedy, Mariyah Sajjad, Michael A. Herrera, Peter Szieber, Natasza Rybacka, Yinan Zhao, Craig Steven, Zainab Alghamdi, Ivan Zlatkov, Julie Hagen, Chloe Lauder, Natalie Rudolfova, Magdalena Abramiuk, Karolina Bolimowska, Daniel Joynt, Angelica Lucero, Gustavo Perez Ortiz, Annamaria Lilienkampf, Alison N. Hulme, Dominic J. Campopiano

**Affiliations:** a School of Chemistry, University of Edinburgh David Brewster Road Edinburgh EH9 3FJ UK Dominic.Campopiano@ed.ac.uk

## Abstract

Organic synthesis often requires multiple steps where a functional group (FG) is concealed from reaction by a protecting group (PG). Common PGs include *N*-carbobenzyloxy (Cbz or Z) of amines and *tert*-butyloxycarbonyl (O^*t*^Bu) of acids. An essential step is the removal of the PG, but this often requires excess reagents, extensive time and can have low % yield. An overarching goal of biocatalysis is to use “green” or “enzymatic” methods to catalyse chemical transformations. One under-utilised approach is the use of “deprotectase” biocatalysts to selectively remove PGs from various organic substrates. The advantage of this methodology is the exquisite selectivity of the biocatalyst to only act on its target, leaving other FGs and PGs untouched. A number of deprotectase biocatalysts have been reported but they are not commonly used in mainstream synthetic routes. This study describes the construction of a cascade to deprotect doubly-protected amino acids. The well known *Bacillus* BS2 esterase was used to remove the O^*t*^Bu PG from various amino acid substrates. The more obscure *Sphingomonas* Cbz-ase (amidohydrolase) was screened with a range of *N*-Cbz-modified amino acid substrates. We then combined both the BS2 and Cbz-ase together for a 1 pot, 2 step deprotection of the model substrate CBz-l-Phe O^*t*^Bu to produce the free l-Phe. We also provide some insight into the residues involved in substrate recognition and catalysis using docked ligands in the crystal structure of BS2. Similarly, a structural model of the Cbz-ase identifies a potential di-metal binding site and reveals conserved active site residues. This new biocatalytic cascade should be further explored for its application in chemical synthesis.

## Abstract 2

A 1 pot, 2 step deprotection biocatalytic cascade.

## Graphical abstract

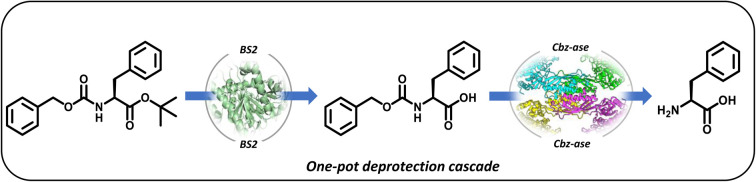

## Introduction

The introduction and subsequent removal of protecting groups (PGs) are often important and widely executed steps in synthetic routes towards complex organic molecules such as peptides, nucleotides, oligosaccharides and natural products. Suitable protecting groups are necessary to prevent the formation of undesired bonds and side reactions. Certainly, a diverse range of protecting groups have been developed for this purpose and chemical methods for both their protection and deprotection are well-established.^1–3^ Regularly, the issue arises where a particular functional group requires selective protection/deprotection in the presence of similarly reactive groups. In such instances, the mildest reaction conditions and highest selectivity are desirable. The application of biocatalysts in protecting group chemistry offers an excellent alternative to chemical methods, with mild reaction conditions and high selectivity as notable advantages. Therefore, it is surprising that biocatalysts are not more commonly used for this purpose.

**Fig. 1:**
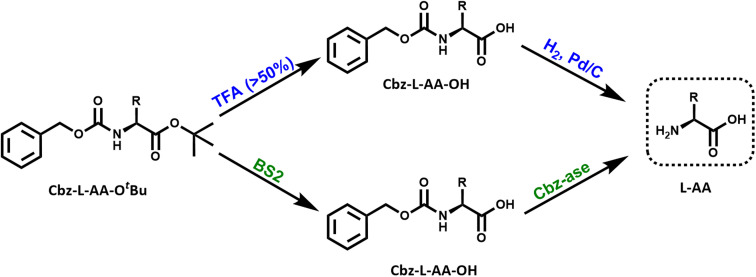
Reaction schemes for the chemical and biocatalytic deprotection of a doubly protected amino acid (Cbz-l-AA-O^*t*^Bu). Top route (blue): treatment with trifluoroacetic acid (% TFA) removes the O^*t*^Bu ester protecting group from the acid that yields the Cbz-protected amino acid. The Cbz group is removed by catalytic hydrogenation over palladium. This results in the free l-amino acid. Bottom route (green): the same sequential reactions are catalysed by the combination of BS2 esterase and Cbz-ase biocatalysts.

Protection of the α-amino group of amino acids in peptide chemistry is important to prevent polymerization of the amino acid once activated.^1^ The first modern protecting group, *N*-carbobenzyloxy (Cbz or Z) group is often used to protect amino groups during organic synthesis.^1^ The deprotection step for this group frequently involves catalytic hydrogenation (Fig. 1). Issues with the selectivity of this method can arise when other reactive functional groups exist in the protected substrate such as carbon–carbon double bonds, thiols or sulfides, which can interfere.^4^ It should also be noted that the toxicity of palladium becomes an issue in the synthesis of peptide drugs. Some examples of enzymatic hydrolysis of Cbz-protected substrates have been reported previously through the use of soil microorganisms,^5^ an immobilized penicillin acylase,^6^ and an amidohydrolase from *Sphingomonas paucimobilis* (Fig. 1).^4^ The latter was reported by Patel *et al.* in which the biocatalyst was isolated from the cell extracts of *S*. *paucimobilis*, and shown to successfully deprotect a number of Cbz-protected amino acids with enantioselectivity for the (l)-enantiomer.^4^ Kinetic resolutions of the d/l-Cbz-substrates resulted in the isolation of both the deprotected l-amino acid and the Cbz-d-amino acid in high ee (>99%).^7^ In subsequent years, it was also reported that a complimentary d-specific Cbz hydrolase existed from *Burkholderia phenazinium*.^8^ Unfortunately, no further characterisation of this potentially useful biocatalyst has been reported. While these studies have highlighted the potential for high activity and selectivity in the deprotection of Cbz-substrates, it is surprising that this class of ‘deprotectases’ have not received more attention in both academic and industrial settings.

Many examples of enzymatic deprotection exist, the literature in this area particularly focuses on carbohydrate protection/deprotection.^9^ However, BS2, an esterase from *Bacillus subtilis* has been shown to deprotect a number of carboxyl protecting groups; the *tert*-butyl group,^10^ benzyl and methyl groups,^11^ and allyl and chloroethyl groups.^12^ The carboxyl protecting groups are also important moieties in peptide chemistry for the protection of the C-terminal carboxylic acid or the side chain carboxylic acid of glutamate or aspartate residues.^1^*tert*-Butyl (^*t*^Bu) protection is often used in both solid and solution-phase peptide synthesis. However, deprotection of this group requires harsh conditions of acid or base,^13^ high temperatures and organic solvents or the use of expensive transition metal complexes.^14^ Acidic conditions (trifluoroacetic acid, TFA) are most commonly employed (Fig. 1), however this may pose a challenge if there are acid-sensitive functional groups present within the protected compound. The use of an enzyme, such as BS2, for the deprotection allows the reaction to occur under mild conditions with high selectivity.

One of the major advantages of biocatalysis is the ability to combine reactions in a one-pot cascade due to the compatibility of biocatalysts with mild conditions and their inherent selectivity.^15^ A cascade is defined as “the combination of at least two chemical steps in a single reaction vessel without the isolation of any intermediate(s)”.^16^ This study aims to further explore two of the existing deprotectases and their combined application in a deprotection cascade to prepare a full deprotected product (Fig. 1). As proof of concept we describe the cloning and characterisation of recombinant BS2 and Cbz-ase and their use in the two-step deprotection of Cbz-l-Phe-O^*t*^Bu to l-Phe.

## Results and discussion

### Enzymatic deprotection of the *N*-carbobenzyloxy (Cbz or Z) group

A synthetic gene encoding the Cbz-ase was ordered from Genscript based on the published sequence.^7^ The original paper reported cloning the gene from *Sphingomonas paucimobilis* SC 16113. However, we could not find an accession code for this, but did find a related amidohydrolase from a *Sphingobium yanoikuyae* strain YC-JY1 (GenBank: QHD65840). The synthetic gene (Fig. S1A†) was cloned in a pET28a plasmid containing an N-terminal 6xhistidine tag to express a recombinant Cbz-ase (447aa, 48 kDa). This plasmid was used to transform *E. coli* BL21 (DE3) cells and the Cbz-ase was overexpressed by addition of IPTG (final conc. 0.2 mM) at O.D. (600 nm) = 0.6. The expression was carried out for 20 h at 16 °C. The cells were harvested by centrifugation and the Cbz-ase was purified by immobilised metal affinity chromatography (IMAC). The enzyme was well expressed and we isolated ∼10 mg litre^−1^ of culture. The recombinant Cbz-ase was analysed by ESI-MS and a molecular weight of 47 930 Da was in good agreement with the predicted mass minus the N-terminal methionine residue (47 927 Da, Fig. S4†). The enzyme was judged to be pure enough for activity assay after IMAC (Fig. S2†) for use in the deprotection of Cbz-l-phenylalanine (Z-l-Phe) and detection of the l-Phe product through a coupled colorimetric assay.^17^ This method uses the FAD-dependent l-amino acid oxidase (l-AAO) and the hydrogen peroxide formed is easily monitored using horseradish peroxidase (HRP) and 2,2′-azino-bis(3-ethylbenzothiazoline-6-sulfonic acid (ABTS), measuring absorbance at 420 nm (Fig. S3†). The purified Cbz-ase was active in this coupled assay (Fig. S5B†) using Z-l-Phe, and the biocatalyst was then tested in the deprotection of various Cbz-protected amino acids by RP-HPLC to detect the formation of the free amino acid (l-Phe, l-Tyr, l-Trp and Gly-l-Phe, Fig. S19–S27,† and Table 1). The hydrolysis of the Cbz group results in the direct formation of both the free amino acid and benzyl alcohol. This direct production of benzyl alcohol allowed the monitoring of deprotection of non-UV active substrates *via* HPLC (*e.g.*l-Ala, l-Lys, l-Pro, l-Glu, Fig. S19–S27† and Table 1).

**Table 1:** Deprotection of Z-amino acids by Cbz-ase

Substrate	Conversion^a^ (%)
Z-l-Phe	100
Z-l-Tyr	100
Z-l-Ala	99
Z-l-Lys	83
Z-l-Gly-Phe	65
Z-l-Trp	7
Boc-l-Lys(Z)-OH	3
Z-l-Glu(O^*t*^Bu)-OH	1
Z-l-Pro	0

a The Z-amino acid substrates (5 mM) and Cbz-ase (0.5 mg mL^−1^) were incubated at 37 °C, 250 rpm for 24 hours. Conversion was determined using HPLC analysis through the quantification of benzyl alcohol.

**Fig. 2:**
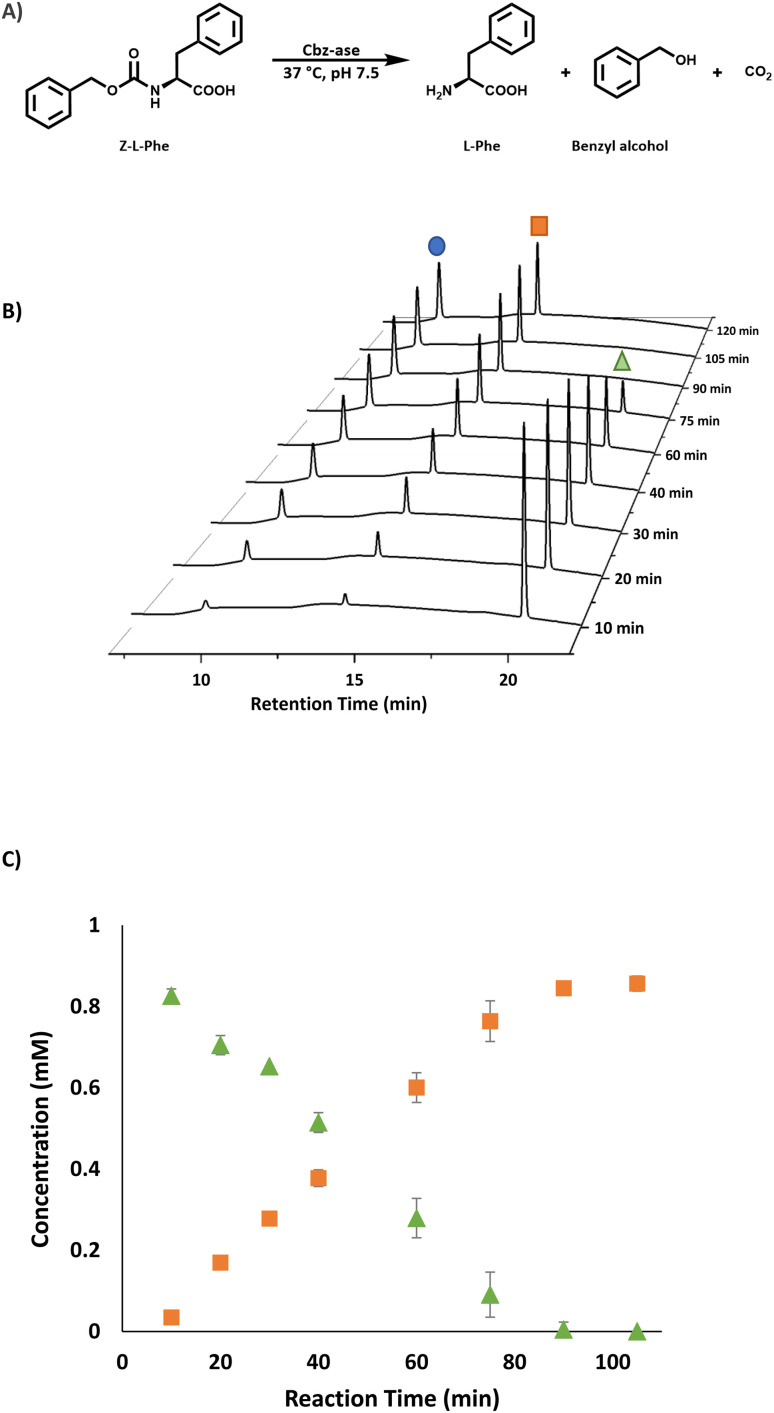
Analysis of the Cbz-ase catalysed reaction. (A) Reaction scheme for the Cbz-ase catalysed deprotection of Z-l-Phe. (B) Reaction monitoring using HPLC analysis showing the formation of l-Phe (9.6 min, blue circle) and benzyl alcohol (14.2 min, orange square) and the disappearance of the Z-l-Phe substrate (20.1 min, green triangle). (C) Plot of concentration (mM) *vs.* reaction time (min) displaying the direct formation of by-product benzyl alcohol (orange square) in relation to the depletion of Z-l-Phe (green triangle). Reaction conditions: Z-l-Phe (10 mM), Cbz-ase (0.075 mg mL^−1^) in sodium phosphate buffer (50 mM, pH 7.5), 1 mL reaction volume, 37 °C, 250 rpm. Samples (100 μL) taken at specified time intervals, quenched with TFA (2 μL, 10% in water) and diluted to 1 mL with water.

The substrate activity we have measured is in good agreement with that reported in the original study by Patel *et al.* (l-Phe, l-Tyr, l-Leu, l-Pro, l-Lys).^7^ They also reported little to no activity with the d-enantiomers of these amino acid substrates although this analysis was carried out with *E. coli* cell extracts expressing the Cbz-ase rather than purified enzyme. HPLC analysis revealed a time-dependent formation of l-Phe (9.6 min) with the depletion of the Cbz-l-Phe substrate (20.1 min, Fig. 2B) and the appearance of the benzyl alcohol by product (14.2 min, Fig. 2B and C). The reaction was complete after 90 min (Fig. 2B and C). The formation of this by-product in the enzymatic hydrolysis differs from the by-product produced in the metal-catalysed hydrogenation that is commonly employed for the deprotection of Cbz groups. The production of benzyl alcohol in enzymatic Cbz-deprotection was also noted by Morozova *et al.* in 2023 through their study of Cbz deprotection by penicillin acylases.^18^

### Enzymatic deprotection of the *tert*-butyl ester group

**Fig. 3:**
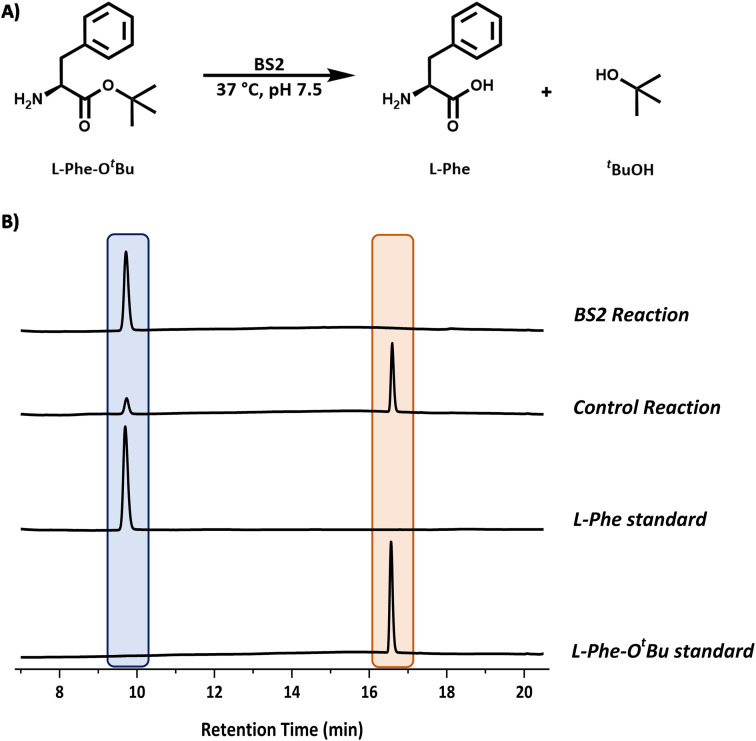
Analysis of the BS2 catalysed reaction. (A) Reaction scheme for the BS2-catalysed deprotection of l-Phe-O^*t*^Bu. (B) Reaction monitoring by HPLC analysis showing the formation of l-Phe (9.6 min, blue) from the l-Phe-O^*t*^Bu substrate (16.6 min, peach). Reaction conditions: l-Phe-O^*t*^Bu (10 mM), BS2 (0.9 mg mL^−1^) in sodium phosphate buffer (50 mM, pH 7.5), 1 mL reaction volume, 37 °C, 250 rpm. Samples (200 μL) taken and quenched with acetonitrile (200 μL) and diluted to 1 mL with water.

We chose the well characterised esterase from *Bacillus subtilis* (BS2) as the biocatalyst for the *tert*-butyl (^*t*^Bu) deprotection of the carboxyl group.^10^ The synthetic gene from Genscript was cloned in a pET28a plasmid containing a C-terminal histidine tag and TEV cleavage site. The sequence (Fig. S1B†) of this clone was based on the *Bacillus subtillis* gene sequence and the PDB code (WP_326228118 and 1QE3). This plasmid was used to transform *E. coli* BL21 (DE3) cells and the protein was overexpressed by the addition of IPTG (final conc. 0.2 mM) at O.D. (600 nm) = 0.6. The expression was carried out for 20 h at 16 °C. The cells were harvested by centrifugation and lysed by sonication. The BS2 was purified by immobilised metal affinity chromatography (IMAC). The enzyme was expressed very well and we isolated ∼45 mg litre^−1^ of culture. The recombinant BS2 (∼56 kDa) was analysed by ESI-MS analysis and a molecular weight of 55 540 Da (Fig. S7†) was in good agreement with the predicted mass minus the N-terminal methionine residue (55 537 Da). The activity of the purified, recombinant BS2 esterase was verified using the *p*-nitrophenolate assay, by monitoring absorbance at 405 nm resulting from the hydrolysis of *p*-nitrophenyl-acetate substrate (Fig. S6†). As reported by Bornscheuer, Kokotos *et al.*, BS2 is capable of hydrolysing a wide range of N-protected *tert*-butyl esters.^10–12^ In accordance with the substrate chosen for the Cbz-ase and for ease of detection by HPLC, l-Phe-O^*t*^Bu was tested as a substrate for BS2. It was shown that BS2 hydrolyses the carboxyl protecting group efficiently with full conversion to form the unprotected l-Phe (9.6 min) from l-Phe-O^*t*^Bu (16.6 min) in 24 h (Fig. 3).

### Biocatalytic cascade for the deprotection of a doubly protected amino acid

**Fig. 4:**
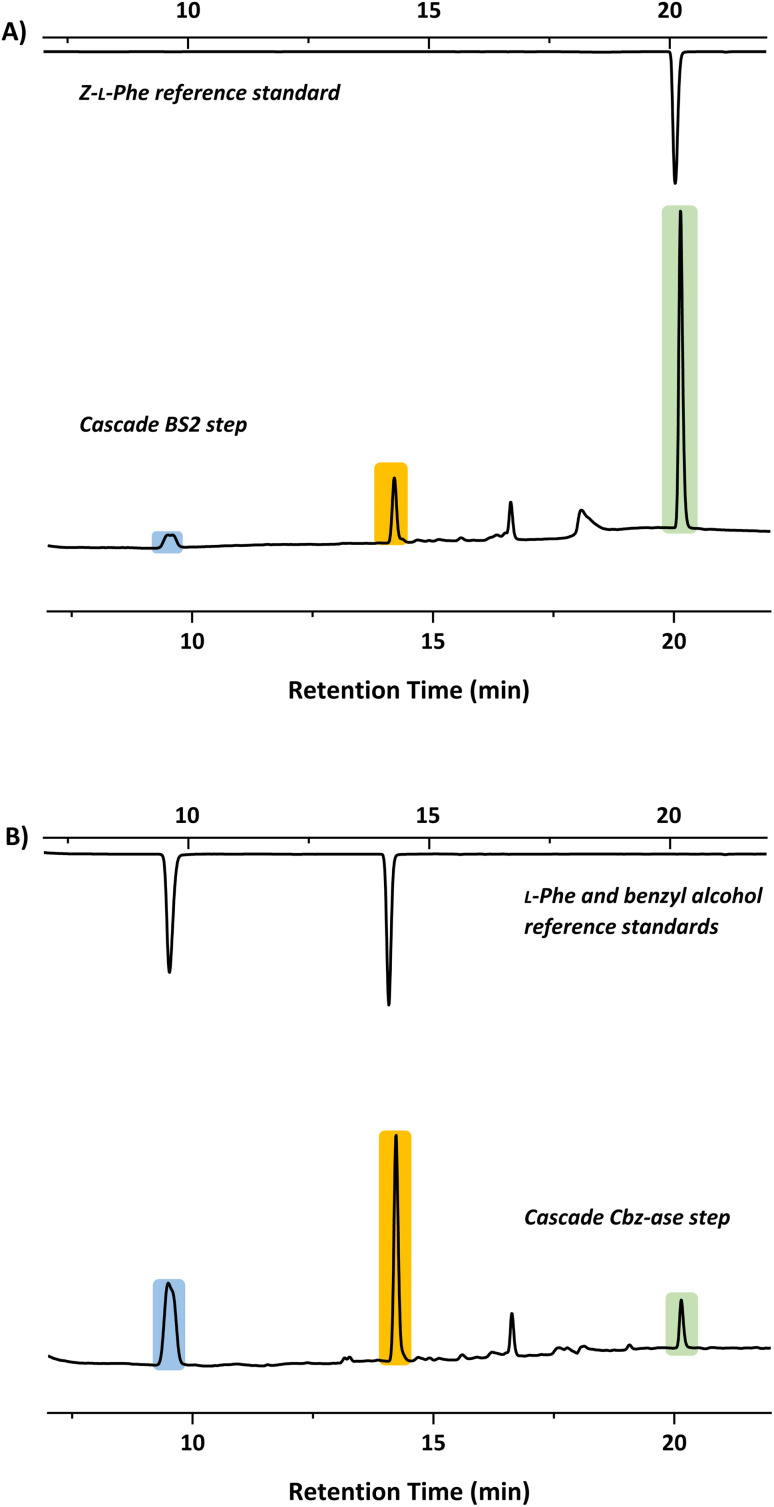
HPLC chromatograms showing product formation after each step of the sequential biocatalytic deprotection cascade. (A) BS2 step in which Z-l-Phe-O^*t*^Bu is deprotected to form Z-l-Phe (green, 20.1 min). (B) Cbz-ase step in which the Z-Phe formed in step A is converted to l-Phe (blue, 9.6 min) and benzyl alcohol (orange, 14.2 min).

In light of the successful deprotection of the singly protected amino acids, it was proposed that a novel one-pot deprotection cascade could be employed for doubly protected substrates. The strategy was to use recombinant BS2 to firstly hydrolyse the *tert*-butyl group, then the recombinant Cbz-ase removes the Cbz group. As a proof of concept, the doubly protected phenylalanine (Z-l-Phe-O^*t*^Bu) was chosen since both biocatalysts had been shown to fully deprotect the corresponding singly-protected l-Phe as described above. The Z-l-Phe-O^*t*^Bu substrate used in the cascade reaction was synthesised in 59% yield using standard methods. The solubility of Z-l-Phe-O^*t*^Bu in the biocatalysis reaction buffer was a challenge, and to prevent precipitation the reaction was performed with low substrate concentration (2 mM), high enzyme loading (2.5 mg BS2, 0.3 mg Cbz-ase) and 10% AcCN to dissolve the substrates. The cascade reaction was carried out in a sequential manner; firstly using BS2 to deprotect the *tert*-butyl group and generate Z-l-Phe over a 24 h period. Despite the low substrate concentration, the disappearance of the Z-l-Phe-O^*t*^Bu was observed. This was accompanied by the appearance of the Z-l-Phe product at 20 min (Fig. 4A). The first step to deprotect Z-l-Phe-O^*t*^Bu with BS2 appeared to not go to completion as some of the doubly-protected Z-l-Phe-O^*t*^Bu substrate was still observed and Z-l-Phe was formed with 26% conversion. The low conversion in this step can somewhat be attributed to the insolubility of Z-l-Phe-O^*t*^Bu, as precipitation could be observed in the reaction. It is also worth noting that the benzyl alcohol by-product was detected suggesting that, under these conditions, BS2 displays some activity in hydrolysing the Cbz group. Then, addition of the Cbz-ase resulted in the deprotection of the Cbz group (over 6 h) from the Z-l-Phe “intermediate” to produce the unprotected l-Phe (at 9.6 min, Fig. 4B) in 89% conversion from the Z-l-Phe intermediate, with 23% overall conversion. The benzyl alcohol by-product was also observed (14.2 min, Fig. 4B). Taken together, this analysis provides supporting evidence that the combination of BS2 with Cbz-ase has produced l-Phe from a doubly-protected amino acid.

### Structural analysis of the BS2 and Cbz-ase biocatalysts

#### BS2

**Fig. 5:**
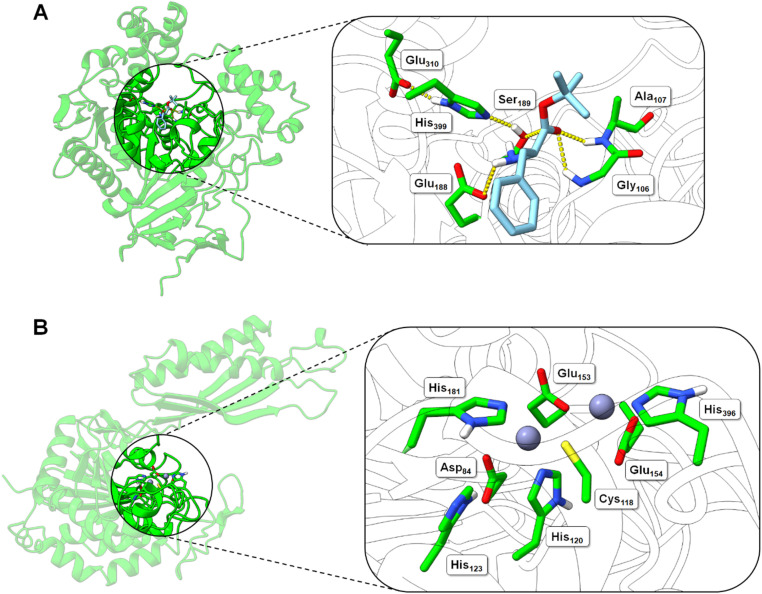
Structural models of the BS2 and Cbz-ase biocatalysts. (A) Model of the BS2 based on the X-ray crystal structure (PDB: 1QE3). The inset shows the putative active with the catalytic triad (Ser_189_, Glu_310_, His_399_) with the key Ser_189_ nucleophile highlighted. The docked l-Phe-O^*t*^Bu ligand is shown to H-bond with the side chains of the potential oxyanion hole (Gly_106_ and Ala_107_) and the amine of the substrate engages in an ionic interaction with Glu_188_. The Phe side chain binds in a hydrophobic pocket. (B) Model of the Cbz-ase structure with two Zn^2+^ ions docked in putative metal-binding pockets. Putative binding residues are shown. A highly conserved Cys_118_ is highlighted and Zn^2+^ ions are shown as grey-blue spheres.

To aid our understanding of the substrate specificity and the activity of the BS2 esterase we took advantage of the extensive studies that have been undertaken with this enzyme. The X-ray structure was originally determined over 25 years ago by Spiller *et al.* (PDB: 1QE3).^19^ BS2 is a member of the α/β hydrolase superfamily which have multiple applications in biocatalysis. The BS2 has many close homologues in the esterase/lipase class of enzymes found in various *Bacillus* species (Fig. S8A†). BS2 is a classic esterase powered by a canonical catalytic triad (Ser_189_, Glu_310_, His_399_), however, it is surprising that (to our knowledge) no ligand-bound structure has been deposited in the PDB. Therefore, to identify residues involved in substrate binding we modelled the l-Phe-O^*t*^Bu into the active site (Fig. 5A).^20^ Across several docking experiments, the docked l-Phe-O^*t*^Bu carbonyl was consistently oriented within a feasible attack distance from the catalytic Ser_189_; this docking experiment enabled the identification of a putative oxyanion hole, formed from the backbone amide hydrogens of Gly_106_ and Ala_107_. The phenyl moiety of l-Phe-O^*t*^Bu is predicted to enter a hydrophobic pocket comprising Leu_67_, Ala_400_ and Cys_404_, and a further hydrogen bond is predicted between the l-Phe-O^*t*^Bu amine and Glu_188_. Whilst the docking experiment was performed using l-Phe-O^*t*^Bu as a model substrate, the estimated dimensions of the BS2 esterase binding pocket (830.4 Å^2^, 695.8 Å^3^) suggests that it is sizeable enough to accommodate the broad substrate range observed for this enzyme (Fig. S9†).^10–12,19^

#### Cbz-ase

It is surprising that the structure of Cbz-ase has not been crystallographically determined, despite being discovered more than 20 years ago. The initial study by the team at Bristol Myers Squibb reported on the purification of the enzyme from *Sphingomas paucimobilis* and its activity against >20 Cbz-protected substrates. This study showed that the enzyme was L-specific. A subsequent paper reported the cloning of the gene and the sequence of the encoded Cbz-ase enzyme. The expression of the recombinant enzyme helped guide our cloning, expression and isolation of the active enzyme we report. After a hiatus of ∼10 years, a follow up report described the isolation of a D-specific Cbz-ase from a *Burkholderia phenazinium* SC 16530 strain. However, no cloning or characterisation of this enzyme has been reported.

In the absence of a crystal structure, we instead built a structural model of *S. paucimobilis* Cbz-ase using the powerful structural prediction tool ColabFold.^21,22^ Cbz-ase is predicted to structurally resemble members of the M20 peptidase family of enzymes (pLDDT = 96.2, pTM = 0.91),^23^ including *N*-α-acylglutamine aminoacylase from *Corynebacterium* sp. (6SLF),^24^ and amidohydrolases from *Staphylococcus aureus* (4EWT),^25^*Bacillus subtilis* (1YSJ) and *Arabidopsis thaliana* (1XMB).^26^ Further homologues were identified in several *Sphingomonas* and *Bacillus* species, with more distant homologues identified in other bacterial phyla by phylogenetic analysis (Fig. S8B†).^27^ The predicted Cbz-ase architecture comprises a 124aa ferredoxin-like “satellite” domain (N_201_–N_324_), which protrudes mid-sequence from the larger α/β/α sandwich fold that defines the catalytic domain (Fig. S10†). It is understood that oligomerisation of M20 peptidases occurs *via* this satellite domain; therefore, by excising and docking this satellite domain against itself, we predict that Cbz-ase assumes a homotetrameric organisation (pTM = 0.96) that closely resembles structural homologues PDB: 4EWT and 6SLF (Fig. S11 and S12†). This is supported by size exclusion chromatography data (Fig. S32†) which is consistent with a homotetrameric structure.

Within the catalytic domain, two metal-binding centres were inferred by structural homology, within which Zn^2+^ could be comfortably docked using the MIB prediction and docking server (Fig. 5B).^28^ The Zn^2+^ ions remain tightly bound in the predicted Cbz-ase structure during a 10 ns molecular dynamics simulation (MDS, Fig. S13†).^29–31^ Of note, the Zn^2+^ ions are coordinated by a strictly-conserved bi-dentate cysteine (Cys_118_) which is also featured in structural homologues of PDB – 4EWT and 6SLF – and sequence homologues identified by evolutionary conservation analysis.^32^ Furthermore, we predict that several highly-conserved acidic (Asp_84_, Glu_153_, Glu_154_) and histidine residues (His_120/123/181/396_) likely contribute to the coordination spheres of the Zn^2+^ ions within the Cbz-ase active site (Fig. S31†). The role(s) of the Zn^2+^ ions in the catalytic mechanism has not been explored for this particular enzyme and further studies should determine if one or both are involved in the hydrolysis of the Cbz group. Our model predicts which residues should be targeted for site directed mutagenesis to probe their role in substrate binding and catalysis.

## Conclusion

The expanded use of biocatalysts in various roles in synthetic chemistry requires well characterised enzymes, available from academic labs, as well as commercial suppliers. For example lipases are used routinely since they are produced on a large scale in highly active forms. Biocatalysts have the potential to be used for removal of commonly used organic protecting groups but their advantages over chemical methods must be emphasised. One such advantage is to be able to couple biocatalysts in a cascade to sequentially remove orthogonal protecting groups. Another is the ability to engineer/evolve a biocatalyst for bespoke synthetic application. Here we describe the combination of a well known BS2 esterase with a more obscure Cbz-ase. Our methods illustrate the ease of application and versatility. In future studies, immobilisation of theses deprotecting enzymes could be carried out to allow for reactions to take place in different solvents and accommodate the solubility of complex substrates. Cascade reactions using natural and engineered biocatalysts are already delivering complex organic molecules at scale.^33^ Moreover, biocatalyst databases such as RetroBioCat are being expanded to construct novel biocatalytic routes to important drug molecules and commodity chemicals.^34,35^

## Experimental

### General

All chemicals and solvents were purchased from Sigma Aldrich or Fisher Scientific and used as received, without further purification. Competent cells were purchased from New England Biolabs. Plasmids were designed and ordered from Genscript.

### High performance liquid chromatography (HPLC)

HPLC analysis was carried out using a Shimadzu instrument fitted with an autosampler (SIL-20A HT), pump (LC-20AD), UV/visible detector (SPD-20A), system controller (CBM-20Alite) and a column oven (CTO-40C). The method for HPLC analysis was as follows; Luna 5 μm C-18 column (250 × 4.6 mm), 30 °C, water (0.1% TFA)/acetonitrile (0.1% TFA) in a gradient at 1 mL min^−1^; 0–3 min 10% acetonitrile (ACN), 3–7 min 20% ACN, 18 min 75% ACN, 21 min 75% ACN, 25 min 10% ACN.

### General method for biocatalyst expression

Commercial *E. coli* BL21 (DE3) competent cells (10 μL) were transformed *via* heat shock transformation with plasmid DNA (2 μL) and selection was carried out on an LB agar plate containing kanamycin (30 μg mL^−1^). The plate was incubated overnight at 37 °C. One colony was used to inoculate a seed culture LB media (250 mL) containing kanamycin (30 μg mL^−1^) and incubated overnight at 37 °C, 200 rpm. The seed culture inoculated LB media (1 L) containing kanamycin (30 μg mL^−1^) to an OD600 of 0.1. The cells were grown at 37 °C, 200 rpm until an OD600 of 0.6–1.0 was achieved. Protein expression was induced using iso-propyl-β-d-1-thiogalactopyranoside (IPTG) (final concentration 0.2 mM), and ZnSO_4_ (final concentration 0.25 mM) was added in the case of Cbz-ase. The temperature lowered to 16 °C, 180 rpm overnight. The cells were harvested by centrifugation (Thermo Scientific Multicentrifuge X3R, 3500*g*, 20 min, 4 °C, 4 × 1000 rotor). The cell pellets were resuspended in phosphate buffer, centrifuged (Thermo Scientific Multicentrifuge X3R, 4000*g*, 45 min, 4 °C, 8 × 50 rotor) and cell pellets were stored at −20 °C.

### General method for biocatalyst purification

The cell pellet was defrosted on ice and resuspended in binding buffer (sodium phosphate (pH 7.4, 50 mM), NaCl (300 mM), imidazole (20 mM)). Cell lysis was carried out by sonication (30 s on 30 s off, 15 cycles) and the cell debris was collected by centrifugation (Thermo Scientific Multicentrifuge X3R, 9000*g*, 50 min, 4 °C, 8 × 50 rotor). The cell lysate was filtered using Millex HA filters (0.45 μm) and loaded onto a pre-equilibrated His Trap nickel affinity column (5 mL) using an ÄKTA explorer (Cytiva Lifesciences, UK) monitoring at 280 nm. The column was washed with 20 column volumes of binding buffer (sodium phosphate (pH 7.4, 50 mM), NaCl (300 mM), imidazole (20 mM)). Elution buffer (sodium phosphate (pH 7.4, 50 mM), NaCl (300 mM), imidazole (300 mM)) were applied with a gradient of 0 to 100% over 10 min, then held at 100% for 10 min, 5 mL min^−1^. Analysis of fractions with high UV/Vis absorbance by 12% SDS-PAGE was carried out. Collected fractions were concentrated and dialysed overnight in dialysis buffer (sodium phosphate (pH 7.4, 50 mM), NaCl (300 mM), 10% glycerol).

### Synthesis of Z-Phe-O^*t*^Bu

The method for synthesis was devised from Strazzolini *et al.*^36^*tert*-Butanol (270 mg, 3.67 mmol, 2.2 eq.), cbz-l-phenylalanine (500 mg, 1.67 mmol, 1 eq.) and 4-dimethylaminopyridine (DMAP, 40 mg, 0.33 mmol, 0.2 eq.) were added to a round-bottom flask. Anhydrous dichloromethane (DCM, 4 mL) was added and the solution was stirred to dissolve the solid reagents at 0 °C. *N*,*N*′-Dicyclohexylcarbodiimide (DCC, 1 g, 4.85 mmol, 2.9 eq.) was added to a beaker and dissolved in anhydrous DCM (3 mL). The DCC solution was cooled in an ice bath before being slowly transferred to the reaction flask. The mixture was stirred in the ice bath for 1 hour, then stirring was continued at room temperature overnight. The mixture was filtered and concentrated under reduced pressure giving a colourless oil. The oil was dissolved in diethyl ether (30 mL) and transferred to a separator funnel. The solution was washed with (i) HCl (0.5 M, 3 × 10 mL), (ii) NaHCO_3_ (5% aq., 3 × 10 mL) and (iii) NaCl (30% aq.). The organic layer was dried over MgSO_4_, filtered and concentrated under reduced pressure. The crude product was purified by column chromatography (hexane/ethyl acetate 9 : 1). Z-Phe-O^*t*^Bu was isolated as a white solid (330 mg, 59% yield).

### Deprotection cascade

A 20 mM stock solution of Z-l-Phe-O^*t*^Bu was prepared in acetonitrile. A solution of purified BS2 (900 μL, 2.8 mg mL^−1^) in sodium phosphate buffer (50 mM, pH 7.5) was prepared. To this, Z-l-Phe-O^*t*^Bu (100 μL, 10 mM) was added in 4 × 25 μL volumes over 4 hours at 37 °C. This was done to prevent precipitation of Z-l-Phe-O^*t*^Bu. A control reaction was also prepared in the same way but without the addition of BS2. The reaction was shaken at 500 rpm, 37 °C for 24 hours. A sample of this reaction was taken (150 μL) and analysed by HPLC. Cbz-ase (500 μL, 1 mg mL^−1^) was added to the reaction and left to react at 500 rpm, 37 °C for 24 hours. The reaction was analysed by reverse-phase HPLC.

## Author contributions

LK and MS carried out biocatalyst purification, assay, chemical synthesis, HPLC analysis and wrote the manuscript. MAH carried out modelling studies and wrote the manuscript. GPO and PS carried out modelling and phylogenetic analysis. NRy, YZ, CS, ZA, IZ, JH, CL, NRu, MA, KB, DJ, ALu carried out the biocatalyst purification, assay, chemical synthesis, HPLC analysis. AL and ANH designed the experiments and analysed data. DJC initiated the project, designed the experiments, analysed data and wrote the manuscript.

## Conflicts of interest

There are no conflicts to declare.

## Supplementary Material

FD-252-D4FD00016A-s001
