# Supplementary material for: Developing deprotectase biocatalysts for synthesis
Source: Faraday Discuss. 2024 Mar 1;252:174–87. doi: 10.1039/d4fd00016a (PMC11389852; doi:10.1039/d4fd00016a)
Supplement: FD-252-D4FD00016A-s001 [file FD-252-D4FD00016A-s001.pdf]

Electronic Supplementary Material (ESI) for Faraday Discussions.
This journal is © The Royal Society of Chemistry 2024

Supplementary Information

Developing Deprotectase Biocatalysts for Synthesis

Lisa Kennedy, Mariyah Sajjad, Michael A. Herrera, Peter Szieber, Natasza Rybacka, Yinan Zhao, Craig Steven, Zainab Alghamdi, Ivan Zlatkov, Julie Hagen, Chloe Lauder, Natalie Rudolfova, Magdalena Abramiuk, Karolina Bolimowska, Daniel Joynt, Angelica Lucero, Gustavo Perez Ortiz, Annamaria Lilienkampf, Alison N. Hulme, Dominic J. Campopiano*

School of Chemistry, University of Edinburgh, David Brewster Road, Edinburgh, EH9 3FJ

Contact: Dominic.Campopiano@ed.ac.uk

Contents

Experimental 3
Materials 3
Analytical Methods 3
NMR spectroscopy 3
Mass spectrometry 3
High Performance Liquid Chromatography (HPLC) 3
Methods 3
Biocatalyst expression and purification 3
Cbz-ase UV/Vis coupled assay 4
Substrate screening Cbzase 4
BS2 *p*-Nitrophenolate assay 5
Hydrolysis of L-Phe-O^{t}Bu by BS2 5
Synthesis of Z-L-Phe-O^{t}Bu 5
Deprotection cascade of Z-L-Phe-O^{t}Bu 6
Computational Methods 7
Design of Phylogenetic Trees 7
BS2 Esterase Molecular Docking using L-Phe-O^{t}Bu 7
Cbz-ase Structural Prediction and Zinc Docking 8
Cbz-ase Evolutionary Conservation Analysis 8
Zinc-docked Cbz-ase Molecular Dynamics Simulation (MDS) 8
Protein sequences 9
Purification of Cbz-ase 10
Cbz-ase activity assay 11
Mass spectrometry analysis of Cbz-ase 12
Purification of BS2 13
BS2 Activity Assay 14
Mass Spectrometry analysis 15
Sequence Analysis 16
Modelling and Docking Studies 18
Spectra 23
NMR 23
HPLC calibration curves 24
HPLC chromatograms 26
References 39

Experimental

Materials

All chemicals and solvents were purchased from Sigma Aldrich or Fisher and used as received, without further purification. Plasmids were ordered from Genscript. *E. coli* competent cells were purchased from New England Biolabs.

Analytical Methods

NMR spectroscopy

^{1}H and ^{13}C NMR spectra were recorded in deuterated chloroform ($CDCl_3$), on a 500 MHz Bruker Spectrometer. The spectra have been referenced with the appropriate residual solvent peaks ($CDCl_3$ 7.26 ppm) and the coupling constants are reported to the nearest 0.1 Hz. Chemical shifts of NMR spectra are reported in parts per million (ppm) on the δ scale. Data are reported in the following way for ^{1}H NMR spectra: chemical shift, integration, multiplicity (s = singlet, d = doublet, t = triplet, q = quartet, and m = multiplet) and coupling constant (*J*) in Hertz (Hz).

Mass spectrometry

Small Molecule Liquid Chromatography-Mass Spectrometry (LC-MS/ESI) was conducted on a Bruker microTOF mass spectrometer with an electrospray ionisation source.

High Performance Liquid Chromatography (HPLC)

HPLC analysis was carried out using a Shimadzu instrument fitted with an autosampler (SIL-20A HT), pump (LC-20AD), UV/visible detector (SPD-20A), system controller (CBM-20Alite) and a column oven (CTO-40C). The method for HPLC analysis was as follows; Luna 5 µm C-18 column (250 x 4.6 mm), 30 °C, water (0.1% TFA)/acetonitrile (0.1% TFA) in a gradient at 1 mL/min; 0 – 3 min 10% acetonitrile (ACN), 3 – 7 min 20% ACN, 18 min 75% ACN, 21 min 75% ACN, 25 min 10% ACN.

Methods

Biocatalyst expression and purification

E. coli BL21 (DE3) competent cells (10 µL) were transformed via heat shock transformation with plasmid DNA (2 µL) and selection was carried out on an LB agar plate containing kanamycin (30 µg/mL). The plate was incubated overnight at 37 °C. One colony was used to inoculate a seed culture LB media (250 mL) containing kanamycin (30 µg/mL) and incubated overnight at 37 °C, 200 rpm. The seed culture inoculated LB media (1 L) containing kanamycin (30 µg/mL) to an OD600 of 0.1. The cells were grown at 37 °C, 200 rpm until an OD600 of 0.6 - 1.0 was achieved. Protein expression was induced using iso-propyl-β-D-1-thiogalactopyranoside (IPTG) (final concentration 0.2 mM). $ZnSO_4$ (final

concentration 0.25 mM) was added in the case of Cbz-ase. The temperature lowered to 16 °C, 180 rpm overnight. The cells were harvested by centrifugation (Thermo Scientific Multicentrifuge X3R, 3500 x g, 20 min, 4 °C, 4 x 1000 rotor). The cell pellets were resuspended in phosphate buffer, centrifuged (Thermo Scientific Multicentrifuge X3R, 4000 x g, 45 min, 4 °C, 8 x 50 rotor) and cell pellets were stored at -20 °C.

The cell pellet was defrosted on ice and resuspended in binding buffer (sodium phosphate (pH 7.4, 50 mM), NaCl (300 mM), imidazole (20 mM)). Cell lysis was carried out by sonication (30 s on 30 s off, 15 cycles) and the cell debris was collected by centrifugation (Thermo Scientific Multicentrifuge X3R, 9000 x g, 50 min, 4 °C, 8 x 50 rotor). The cell lysate was filtered using Millex HA filters (0.45 µm) and loaded onto a pre-equilibrated His Trap Nickel affinity column (5 mL) using an ÄKTA explorer (Cytiva Lifesciences, UK) monitoring at 280 nm. The column was washed with 20 column volumes of binding buffer (sodium phosphate (pH 7.4, 50 mM), NaCl (300 mM), imidazole (20 mM)). Elution buffer (sodium phosphate (pH 7.4, 50 mM), NaCl (300 mM), imidazole (300 mM)) was applied with a gradient of 0 to 100% over 10 min then held at 100% for 10 min, 5 mL/min. Analysis of fractions with high UV/Vis absorbance by 12% SDS-PAGE was carried out. Collected fractions were concentrated and dialysed overnight in dialysis buffer (sodium phosphate (pH 7.4, 50 mM), NaCl (300 mM), 10% glycerol). The purified biocatalyst was aliquoted, flash frozen in liquid nitrogen and stored at -80 °C.

If required, the protein was further purified using size exclusion chromatography prior to freezing using a Superdex HiLoad 16/60 S200 column in storage buffer (sodium phosphate (pH 7.4, 50 mM), NaCl (300 mM), 10% glycerol). Fractions containing the protein were collected, concentrated and frozen at at -80 °C.

Cbz-ase UV/Vis coupled assay

The assay was adapted from Sánchez-Carrón *et al.*, 2015.[1] To a cuvette Z-L-Phenylalanine (150 µL, 20 mM), horse radish peroxidase (10 µL, 100 U/mL), ABTS (10 µL, 50 mM) and L-amino acid oxidase (10 µL, 10000 U/mL) were added. Sodium phosphate buffer (696 µL, 50 mM, pH 7.4) was added to make the volume up to 1 mL. The UV/vis spectrometer, set to 37°C, was blanked using this cuvette before adding Cbz-ase (124 µL, 1.69 mg/mL) and monitoring the absorbance at 420 nm over 12 minutes.

Substrate screening Cbzase

Stocks of substrates were prepared in reaction buffer to a final concentration of 20 mM. Those substrates that are less soluble in water were dissolved in 20% DMSO in buffer to a final concentration of 20 mM (Z-Tyr, Z-Trp, Z-Glu(O^{t}Bu)). The Cbz-protected substrate (250 µL, 20 mM) and Cbzase (295 µL, 1.69 mg/mL) were added to an Eppendorf (1.5 mL). Reaction buffer (455 µL, sodium phosphate 50 mM, pH 7.5) was added to reach a final volume of 1 mL. The reactions were left at 37 °C, 250 rpm for

24 hours. A sample (500 µL) of the reaction was taken and trifluoroacetic acid (TFA, 10 µL, 10% in water) was added before centrifuging on a benchtop centrifuge (13000 xg, 10 min). The supernatant (250 µL) was diluted into water (750 µL) and analysed by reverse-phase HPLC using the method described above.

BS2 *p*-Nitrophenolate assay

A solution of *p*-Nitrophenyl acetate (1 mM) was prepared in ethanol. The UV/vis spectrometer was blanked with sodium phosphate buffer (50 mM, pH 8). To a cuvette, sodium phosphate buffer (965 µl, 50 mM, pH 8), *p*-Nitrophenyl acetate (25 µl, 1 mM) and BS2 (10 µl, 0.5mg/ml) were added. The absorbance over time was monitored at 405 nm.[2]

Hydrolysis of L-Phe-O^{t}Bu by BS2

Reactions were prepared in triplicate. H-Phe-O^{t}Bu (200 µL, 100 mM) and BS2 (125 µL, 7.1 mg/mL) were added to an Eppendorf tube (1.5 mL) and the volume was made up to 1 mL with reaction buffer (sodium phosphate 50 mM, pH 7.5). Control reactions were also prepared in the same way without addition of BS2. The reactions were placed at 37 °C, 250 rpm for 24 hours. The reaction was centrifuged on a benchtop centrifuge (10,000 rpm, 10 min) and the supernatant (200 µL) was removed, diluted to 2 mL with HPLC grade water and analysed by reverse-phase HPLC using the method described above.

Synthesis of Z-L-Phe-O^{t}Bu

The method for synthesis was devised from Strazzolini et al.[3]

tert-Butanol (270 mg, 3.67 mmol, 2.2 eq.), cbz-phenylalanine (500 mg, 1.67 mmol, 1 eq.) and 4-dimethylaminopyridine (DMAP, 40 mg, 0.33 mmol, 0.2 eq.) were added to a dry round-bottom flask. Anhydrous dichloromethane (DCM, 4 mL) was added and the solution was stirred to dissolve the solid reagents at 0 °C. N, N'-Dicyclohexylcarbodiimide (DCC, 1 g, 4.85 mmol, 2.9 eq.) was added to a dry beaker and dissolved in anhydrous DCM (3 mL). The DCC solution was cooled in an ice bath before being slowly transferred to the round-bottom flask. The mixture was stirred in the ice bath for 1 hour, then continued stirring at room temperature overnight. The mixture was filtered and concentrated under reduced pressure giving a colourless oil. The oil was dissolved in diethyl ether (30 mL) and transferred to a separatory funnel. The solution was washed with (i) HCl (0.5 M, 3 x 10 mL), (ii) NaHCO3 (5% aq., 3 x 10 mL) and (iii) NaCl (30% aq.). The organic layer was dried over MgSO4, filtered and concentrated under reduced pressure. The crude product was purified by column chromatography (hexane/ethyl acetate 9:1).

Characterisation: Z-L-Phe-O^{t}Bu was isolated as a white solid (330 mg, 59% yield). ^{1}H-NMR: (500 MHz, $CDCl_3$) δ 7.38 – 7.23 ppm (m, 8H), δ 7.18 – 7.14 (d, *J* = 7.0 Hz, 2H) 5.25 (br, d, *J* = 8.2 Hz, 1H) δ 5.14 (d, *J* = 12.3 Hz, 1H) δ 5.10 ppm (d, *J* = 12.3 Hz, 1H), δ 4.52 ppm (dt, *J* = 6.1, 8.2 Hz, 1H) δ 3.08 (d, *J* = 6.1 Hz, 2H) δ 1.39 (s, 9H). ^{13}C-NMR: (125 Hz, $CDCl_3$) δ 170.4 ppm, δ 155.5 ppm, δ 136.3 ppm, δ 136.0 ppm, δ 129.4 ppm, δ 128.4 ppm, δ 128.3 ppm, δ 128.0 ppm, δ 127.9 ppm, δ 126.8 ppm, δ 82.2 ppm, δ 66.7 ppm, δ 55.1 ppm, δ 38.3 ppm, δ 27.8 ppm. LC-MS (ESI) (*m/z*): Calculated $C_{21}H_{25}NO_4$ $[M + Na]^+$: Predicted 378.16868, found: 378.2. $C_{42}H_{50}N_2O_8$ $[2M + Na]^+$: 733.3372, found: 733.4. Data in line with published literature.[4]

Deprotection cascade of Z-L-Phe-O^{t}Bu

A 10 mM stock solution of Z-L-Phe-O^{t}Bu was prepared in acetonitrile. A solution of purified BS2 (900 µL, 2.8 mg/mL) in sodium phosphate buffer (50 mM, pH 7.5) was prepared. To this, Z-L-Phe-O^{t}Bu (100 µL, 10 mM) was added in 4 x 25 µL volumes over 4 hours at 37 °C. This was done to prevent precipitation of Z-Phe-O^{t}Bu. A control reaction was also prepared in the same way but without the addition of BS2. The reaction was placed at 37 °C, 500 rpm for 24 hours. A sample of this reaction was taken (200 µL), diluted with acetonitrile (100 µL) to ensure dissolution of reaction components, centrifuged on a benchtop centrifuge (10,000 rpm, 5 min) and analysed by RP-HPLC. Cbz-ase (400 µL, 1 mg/ml) was added to the remainder of the reaction (800 µL) and left to react at 37 °C, 500 rpm for 6 hours and analysed in the same way.

Computational Methods

Design of Phylogenetic Trees

BS2:

A small database of 200 proteins with sequence identity to BS2 ranging from 39-99% for PDB/Uniprot, RefSeq and NR database with Blastp was generated. Sequence alignment was performed via Mega7 using ClustalW algorythim (*Pairwise alignment: Gap opening penalty = 10, Gap extension penalty = 0.10; Multiple Gap opening penalty = 10, Gap extension penalty = 0.20*). A phylogenetic tree of aligned proteins was generated with Mega7 using Neighbor-joining tree with bootstrap method. Phyla of the 3 bacteria species expressing the 134 proteins were affirmed via DSMZ: BacDive database. The generated phylogenic tree was designed with ITOL v6 (Fig. S8A).

Cbz-ase:

A small database of 200 proteins with sequence identity to Cbz-ase ranging from 25-40% for PDB/Uniprot databases and >85% for NR database with Blastp was generated. Sequence alignment was performed via Mega7 using ClustalW algorythim (*Pairwise alignment: Gap opening penalty = 10, Gap extension penalty = 0.10; Multiple Gap opening penalty = 10, Gap extension penalty = 0.20*). A phylogenetic tree of 36 aligned proteins was generated with Mega7 using Neighbor-joining tree with bootstrap method. Phyla of the 5 bacteria species expressing the 36 proteins were affirmed via DSMZ: BacDive database. The generated phylogenic tree was further designed with ITOL v6 to achieve the final figure (Fig. S8B).

BS2 Esterase Molecular Docking using L-Phe-O^{t}Bu

The structure of BS2 esterase was reproduced using ColabFold (v.1.55)[5, 6] via AlphaFold2.ipynb, configured to utilise PDB: 1QE3_A as a template model to repair missing residues (RMSD <0.15 Å, pLDDT = 96.7, pTM = 0.960). Steric clashes were subsequently eliminated by AMBER relaxation. The BS2 esterase binding site was identified by topological analysis via the CASTp 3.0 server, using a probe radius of 1.4 Å. Both the L-Phe-O^{t}Bu ligand and BS2 esterase receptor were prepared using AutoDockTools (v.1.5.7), and ligand docking was performed using AutoDock Vina (v1.1.2). The docking experiment was repeated up to 10 times with gradually increasing exhaustiveness (8-128). The top-ranked pose from each docking experiment was sampled for visualisation and analysis in UCSF ChimeraX (v1.7)[7] and PyMOL (v2.5.4). The top-ranked L-Phe-O^{t}Bu poses were virtually identical in their overall orientation, with a computed binding affinity of -6.0 kcal mol^{-1}.

Cbz-ase Structural Prediction and Zinc Docking

All structural predictions were performed using ColabFold (v.1.55)[5, 6] via AlphaFold2.ipynb. In brief, a deep multiple sequence alignment (MSA) was generated using MMSeqs2 prior to structure prediction using AlphaFold 2. Additional structural templates, sourced from the PDB, were utilised for prediction. When appropriate, ColabFold was configured to perform homomeric prediction. The output of the AlphaFold 2 structure module was recycled up to 3 times for refinement. A total of 5 models was generated and ranked by Predicted Template Model score (pTM); Predicted Local Distance Difference Test (pLDDT) scores were also computed for each model to evaluate fold-level confidence. The best model was subsequently relaxed to eliminate steric clashes, and Zn^{2+} ions were docked using the Metal Ion-Binding Site Prediction and Docking Server.[8] Visual inspection was performed in UCSF ChimeraX (v1.7)[7] and PyMOL (v2.5.4).

Cbz-ase Evolutionary Conservation Analysis

Evolutionary conservation analysis was performed using the ConSurf[9] server configured to build MSAs using MAFFT. Up to 150 homologous sequences with identities ranging from 30-95% were compiled from UNIREF90 using the HMMER search algorithm. Conservation scores were calculated via the Bayesian method.

Zinc-docked Cbz-ase Molecular Dynamics Simulation (MDS)

Simulations were performed using GROMACS 2021.4. Protein and ion charges were computed using CHARMM36 all-atom forcefield.[10] Cys_{118} was specially parameterised for Zn^{2+} coordination as per the CHARMM36 residue database used for pdb2gmx. The model was solvated in TIP3P water in a cubic box, and the net protein charge was counterbalanced using simulated sodium ions. The system was energy-minimised by sequential steepest descent/conjugate gradient descent and equilibrated to 300 K and 1 bar using V-Rescale thermostat/Berendsen barostat. Following a 10 ns (5×10^6 time steps) production MD, the trajectory was recentred with additional rotational and translational fitting. Further analysis was performed in GROMACS using gmx gyrate and gmx rms. UCSF Chimera 1.16 was used for trajectory visualisation and for computing pairwise RMSDs.

Protein sequences

(A) Cbz-ase sequence:

```
  1   MGSSHHHHHH SSGENLYFQG HMVQPTPTPQ SELAGLIARD MEGLMTLYRD
 51   LHANPELSLQ EANTAAKLAK RLKAMKFDVT EKVGGTGVVA VMKNGSGPVL
101   LIRADMDGLP VVEQTGLDFA SKVRTKTPEG VETGVMHACG HDTHMTAFIE
151   TAKLLSSQKD KWKGTLVMIL QPAEEVGKGA RDMLEDGLYT RFPRPTHAIA
201   FHDAANLQAG VVGYTPGYAL ANVDSVDIVV KGLGGHGAYP QTTRDPIVLG
251   SRIVTSLQTL VSREQDPQDP AVVTVGSFQA GAKHNIIPDQ ALLLLTVRSY
301   SDETRAKLIK GIERIARGEA IAAGVPDDKM PVISVKDEFT PSTYNPPEFA
351   EQMGALLKGH FAEGRVVKTP AVMGGEDFGR FYRADKSINS FIFWVGGVPA
401   DKMAAAEAGQ ITLPSLHSPF WAPEADKVIA TASEAMTVLA MDILKKD
```

Sequence length 447aa   Predicted Mol Weight. 48061.95 Da

(B) BS2 sequence:

```
  1   MTHQIVTTQY GKVKGTTENG VHKWKGIPYA KPPVGQWRFK APEPPEVWED
 51   VLDATAYGPI CPQPSDLLSL SYTELPRQSE DCLYVNVFAP DTPSQNLPVM
101   VWIHGGAFYL GAGSEPLYDG SKLAAQGEVI VVTLNYRLGP FGFLHLSSFD
151   EAYSDNLGLL DQAAALKWVR ENISAFGGDP DNVTVFGESA GGMSIAALLA
201   MPAAKGLFQK AIMESGASRT MTKEQAASTA AAFLQVLGIN ESQLDRLHTV
251   AAEDLLKAAD QLRIAEKENI FQLFFQPALD PKTLPEEPEK SIAEGAASGI
301   PLLIGTTRDE GYLFFTPDSD VHSQETLDAA LEYLLGKPLA EKAADLYPRS
351   LESQIHMMTD LLFWRPAVAY ASAQSHYAPV WMYRFDWHPE KPPYNKAFHA
401   LELPFVFGNL DGLERMAKAE ITDEVKQLSH TIQSAWITFA KTGNPSTEAV
451   NWPAYHEETR ETVILDSEIT IENDPESEKR QKLFPSKGEE NLYFQGHHHH
501   HH
```

Sequence length 502 aa   Predicted Mol Weight. 55668.73 Da

Figure S1. Protein sequences of recombinant biocatalysts. (A) The Cbz-ase sequence is from *Sphingomonas yanoikuyae* (GenBank: MBO9525196). (B) The *Bacillus subtilis* (strain 168) BS2 sequence is based on the Uniprot sequence: P37967, GenBank: U06089.1 and the PDB code 1QE3.

Purification of Cbz-ase

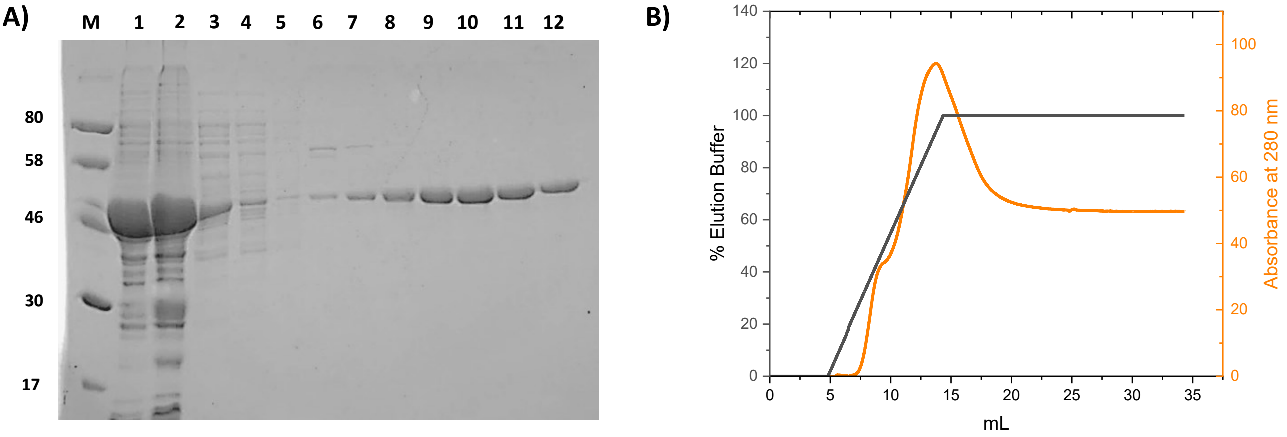

Figure S2. Purification of Cbz-ase. A) SDS-gel of Cbz-ase purification; M = molecular weight marker, 1 = cell pellet, 2 = cell lysate, 3 - 4 = flow through, 5 – 12 = IMAC fractions. B) Chromatogram from Cbz-ase IMAC purification.

Cbz-ase activity assay

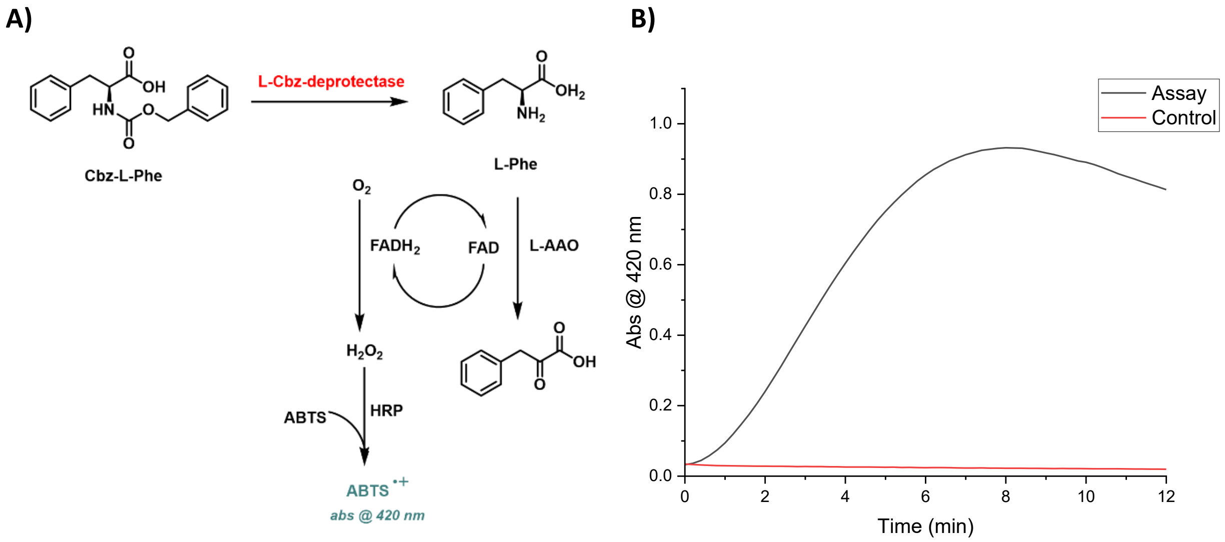

Figure S3. Activity assay for purified Cbz-ase. A) Reaction scheme for the coupled L-AAO and HRP assay for Cbz-ase activity. L-AAO = L-amino acid oxidase, FAD = flavinadenine dinucleotide, HRP = horse radish peroxidase, ABTS = 2,2'-Azino-bis(3-ethylbenzothiazoline-6-sulfonic acid). B) Absorbance at 420 nm over time during activity assay. Assay conditions: Z-L-Phenylalanine (150 µL, 20 mM), horse radish peroxidase (10 µL, 100 U/mL), ABTS (10 µL, 50 mM), L-amino acid oxidase (10 µL, 10000 U/mL) and sodium phosphate buffer (696 µL, 50 mM, pH 7.4) were added to a cuvette. The UV/vis spectrometer was blanked using this cuvette before adding Cbz-ase (124 µL, 1.69 mg/mL) and monitoring the absorbance at 420 nm over 12 minutes.

Mass spectrometry analysis of Cbz-ase

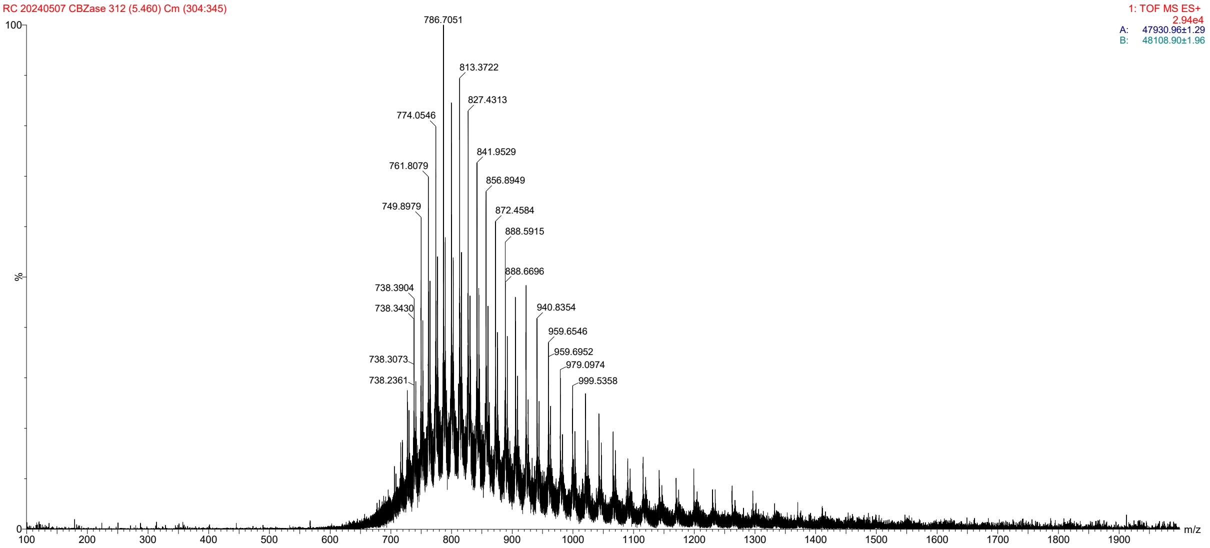

Figure S4. Electrospray ionisation (ESI) analysis. Mass spectrum of purified Cbz-ase. Predicted mass; 48,062 Da (without N-term Met residue = 47,927 Da). Calculated masses 47,930, 48,109 Da.

Purification of BS2

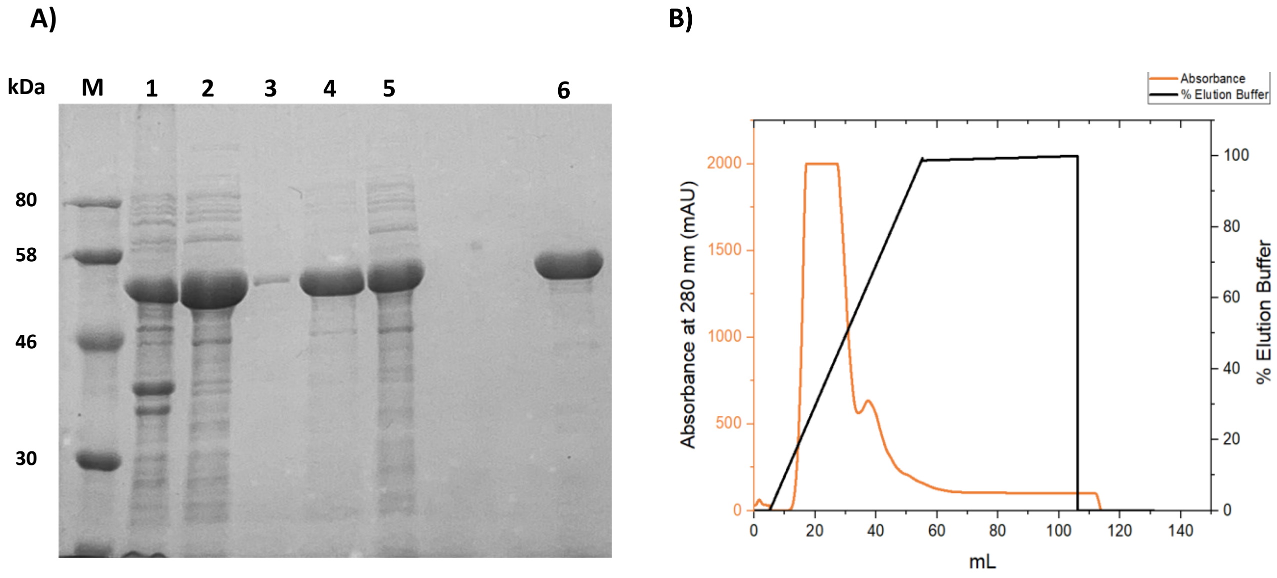

Figure S5. Purification of BS2. A) SDS gel of BS2 puification; M = molecular weight marker, 1 = cell pellet, 2= cell lysate, 3 – 4 = fractions from IMAC, 5 = flow through IMAC, 6 = purified protein after dialysis. B) Chromatogram from IMAC purification of BS2.

BS2 Activity Assay

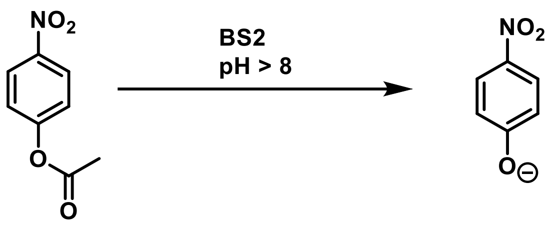

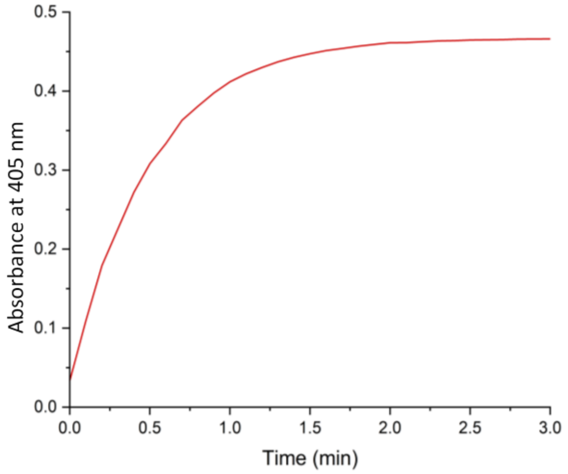

Figure S6. Activity assay of BS2. The hydrolysis of the *p*-nitrophenyl acetate substrate is monitored by the appearance of the *p*-nitrophenolate product 405 nm. Assay conditions: To a cuvette, sodium phosphate buffer (965 µl, 50 mM, pH 8), *p*-Nitrophenyl acetate (25 µl, 1 mM) and BS2 (10 µl, 0.5mg/ml) were added. The absorbance over time was monitored at 405 nm.

Mass Spectrometry analysis

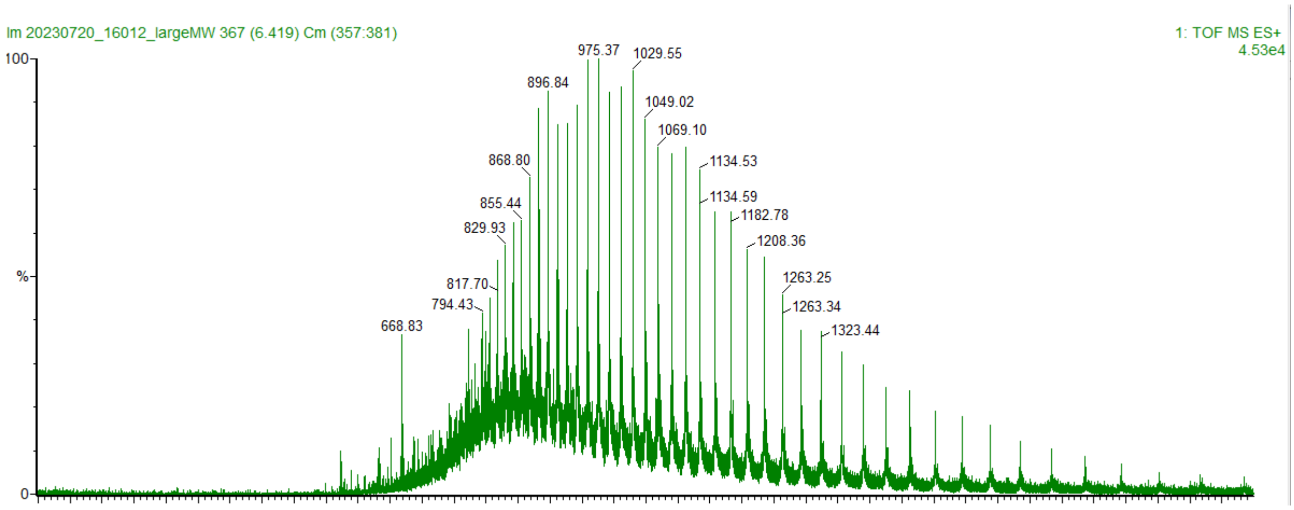

Figure S7. Electrospray ionisation (ESI) analysis. Mass spectrum of purified BS2. Predicted mass; 55,672.78 (without N-term Met residue = 55,537 Da). Calculated masses 55,540 Da, 55,670 Da.

Sequence Analysis

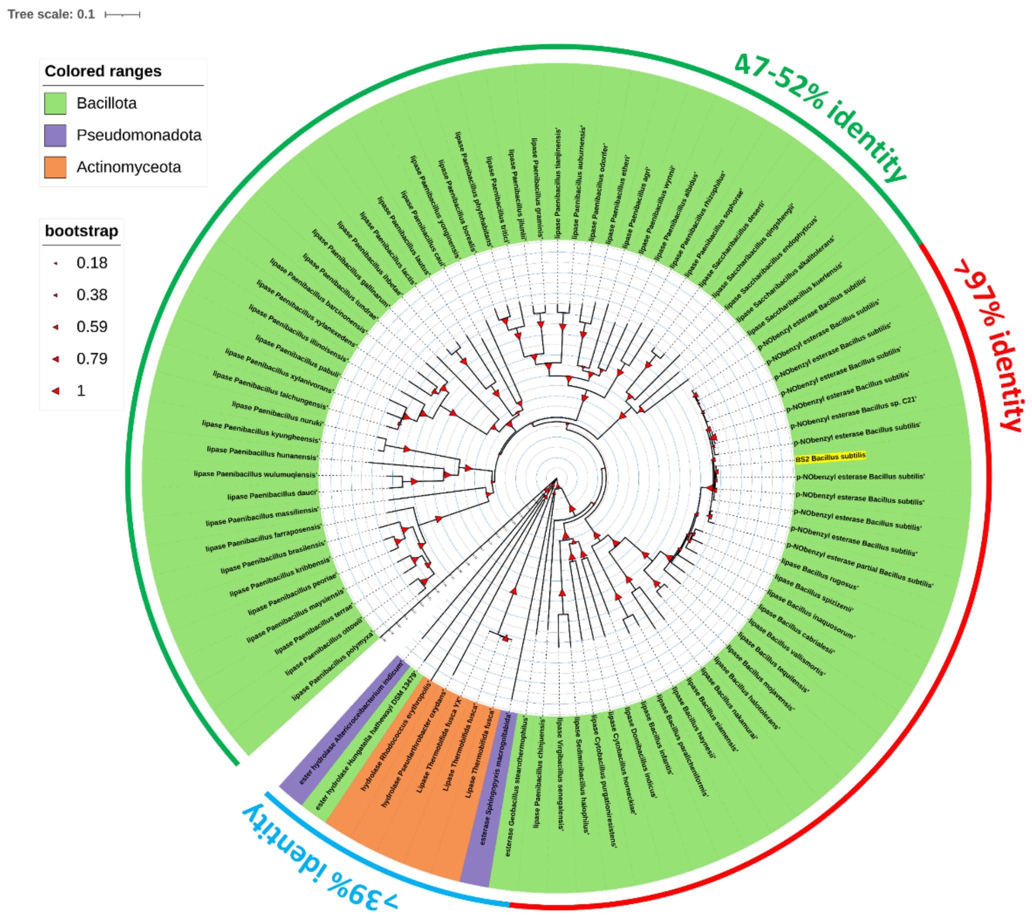

Figure S8A. Phylogenetic analysis of BS2 and its homologues shows a large variation of sequence identity amongst the different phyla of bacteria, even within the same families. The highest sequence identity enzymes compared to BS2 are all found in *Bacillus.*

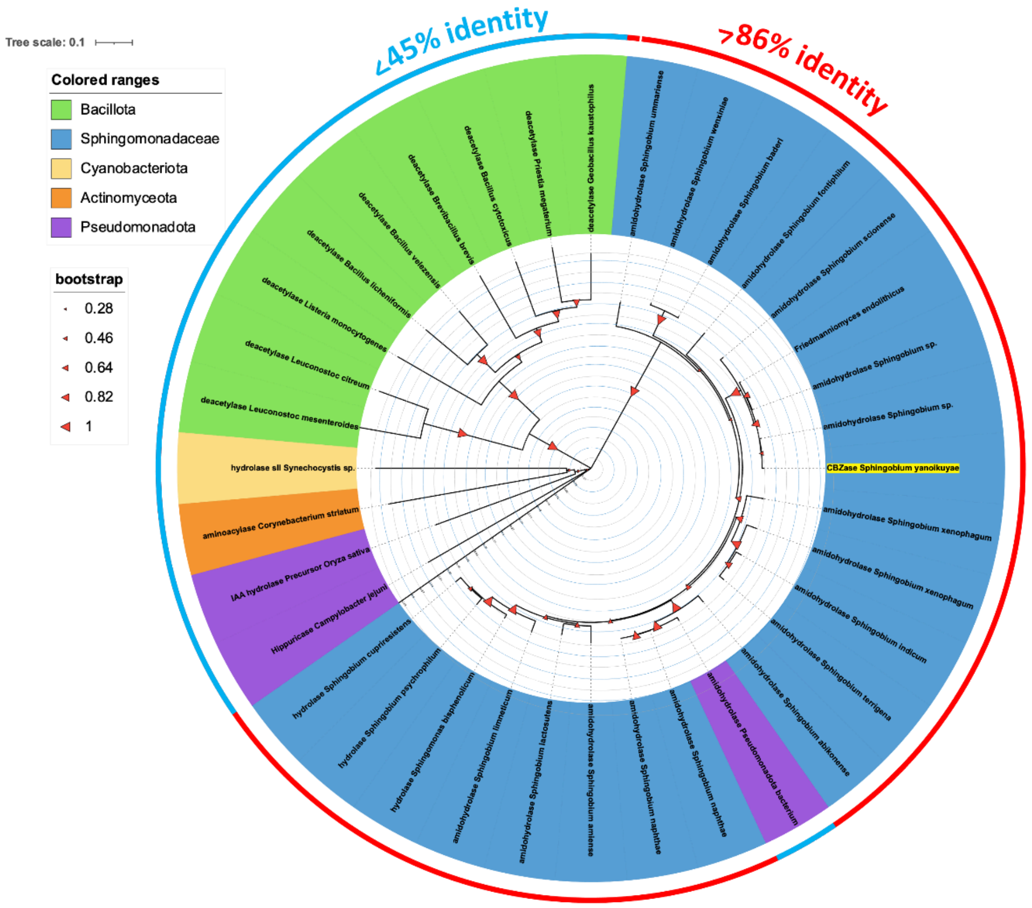

Figure S8B. Phylogenetic analysis of CBZase representing a small variety of bacteria endogenously expressing CBZase-alike enzymes. CBZase from (insert actual strain) shows highest of sequence identity of amidohydrolases from *Sphingomonadaceae.*

Modelling and Docking Studies

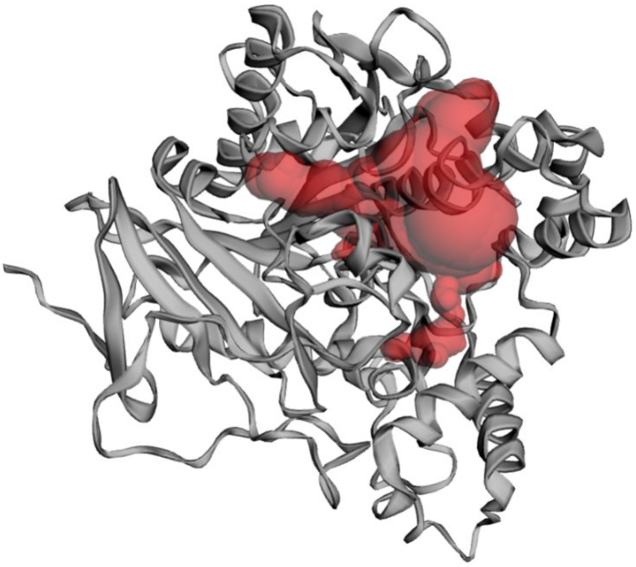

Figure S9. The predicted binding pocket of BS2 esterase, with a computed surface area and volume of 830.4 Å^2 and 695.8 Å^3, respectively. Topological analysis was performed using the CASTp 3.0 server.[11]

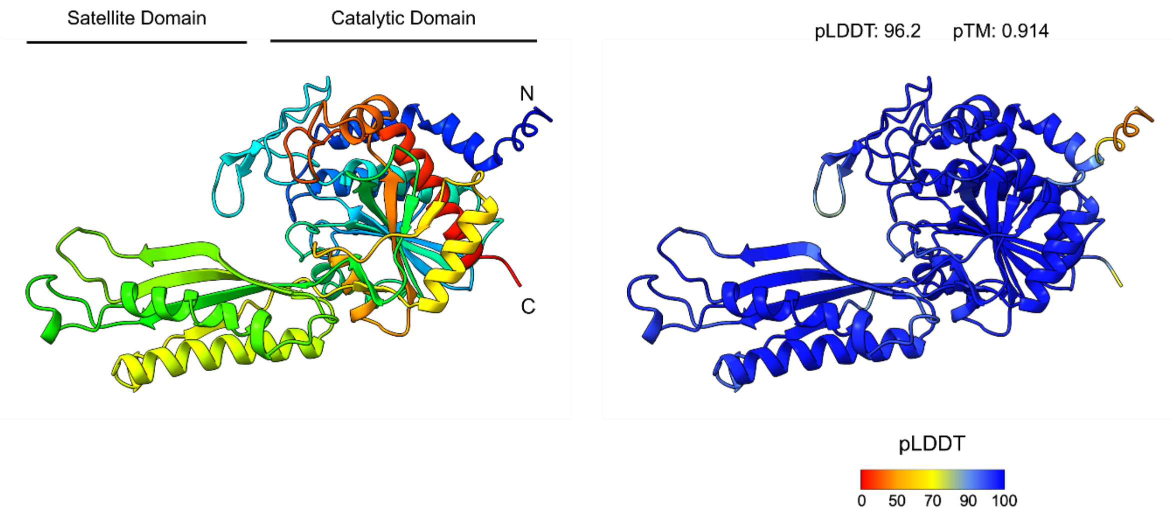

Figure S10. Annotated ColabFold-predicted tertiary structure of Cbz-ase, coloured by rainbow (left) and pLDDT (right).

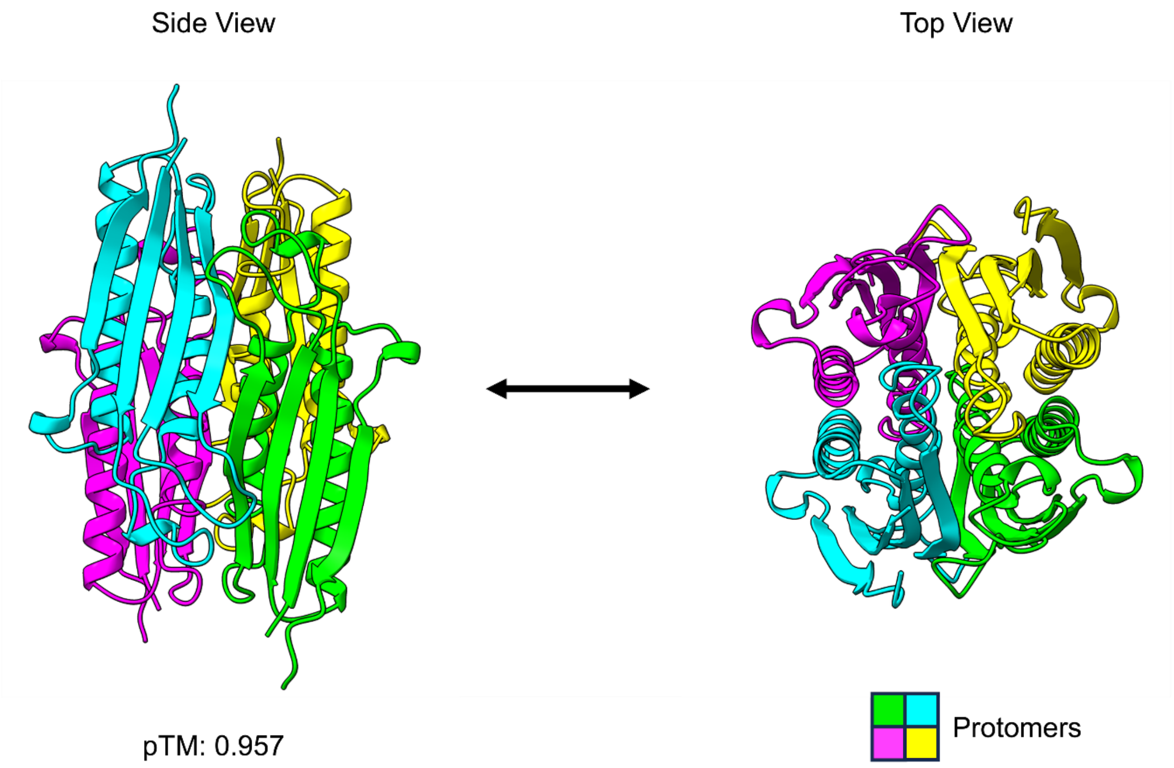

Figure S11. The predicted homotetrameric organisation of the Cbz-ase satellite domains.

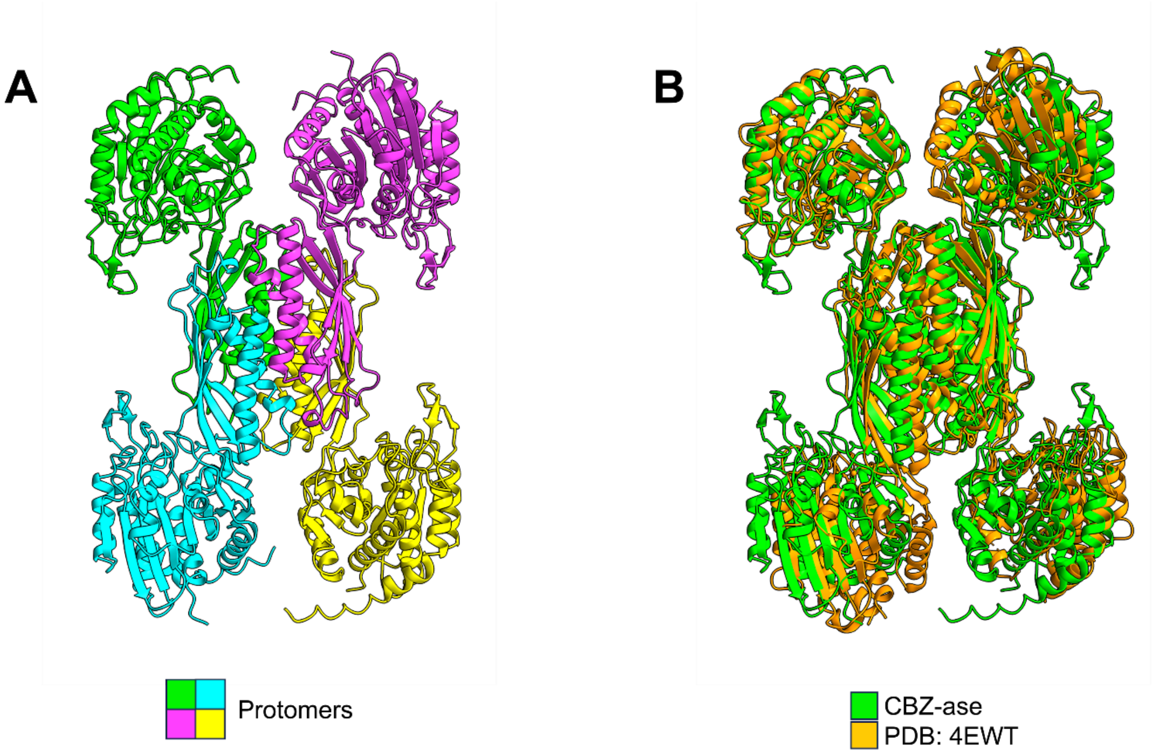

Figure S12. A) A composite homotetrameric model of Cbz-ase, showing the relative organisation of the satellite and catalytic domains of each protomer. **B)** The Cbz-ase homotetramer superimposed on the crystal structure of an amidohydrolase homologue from *Staphylococcus aureus* (PDB: 4EWT).[12]

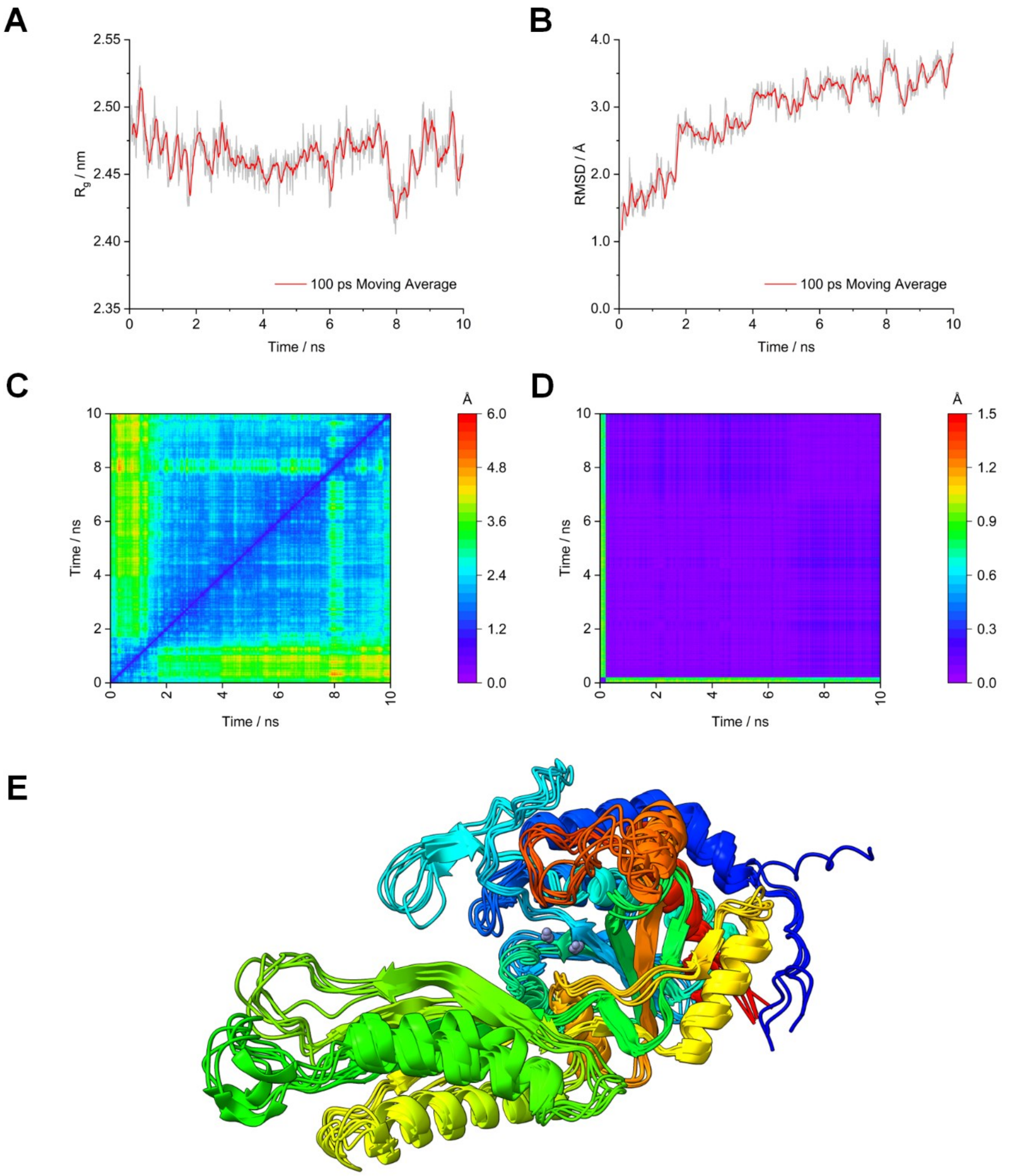

Figure S13. A graphical summary of the Zn^{2+}-docked Cbz-ase MDS. **A)** The radius of gyration (R_g) of the Zn^{2+}-docked Cbz-ase over time. **B)** A 1D RMSD plot (relative to t = 0 ns) of the Zn^{2+}-docked Cbz-ase over time. **C)** A pairwise (2D) RMSD map computed between Cbz-ase Cα atoms at every time point of the MDS. **D)** A pairwise (2D) RMSD map computed between the Zn^{2+} ions at every time point of the MDS. **E)** Cbz-ase morphology over time; frames were extracted at t = 0, 2.5, 5, 7.5 and 10 ns. The docked Zn^{2+} ions are depicted as blue-grey spheres.

Spectra

NMR

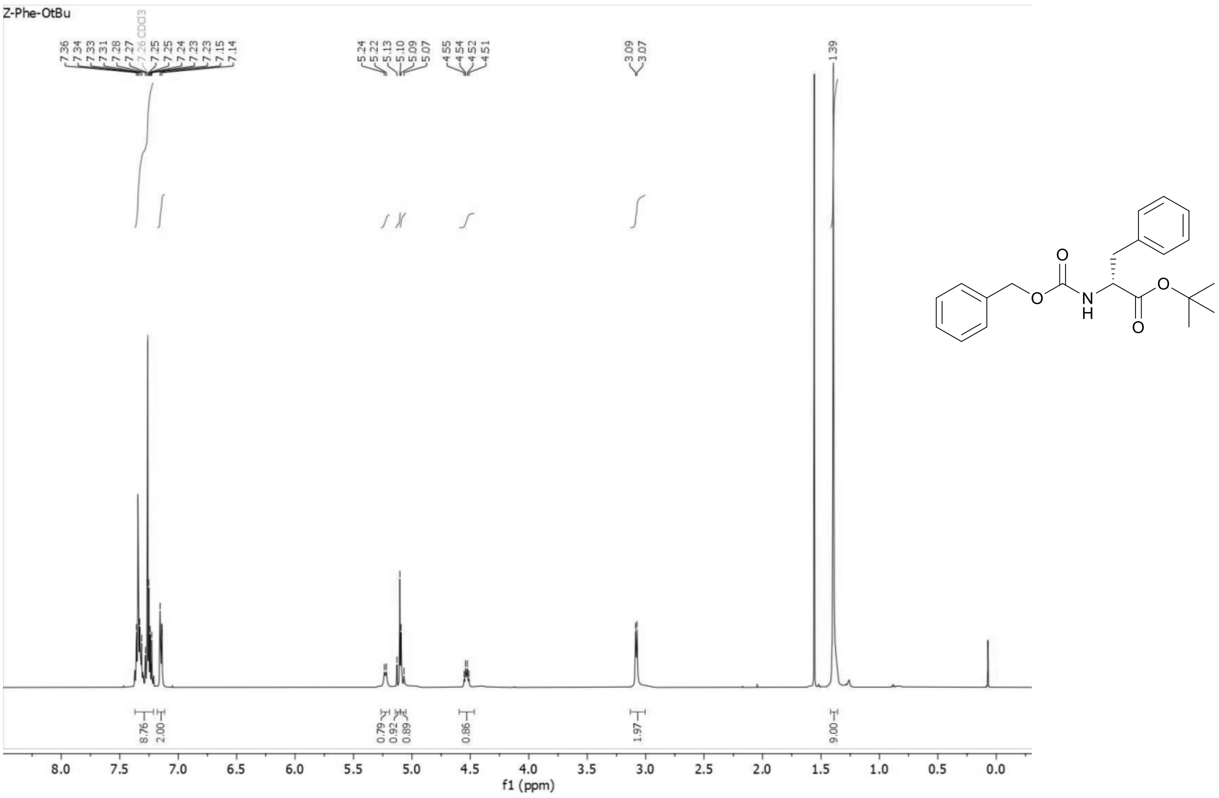

Figure S14. ^{1}H-NMR spectrum of synthesised Z-Phe-O^{t}Bu.

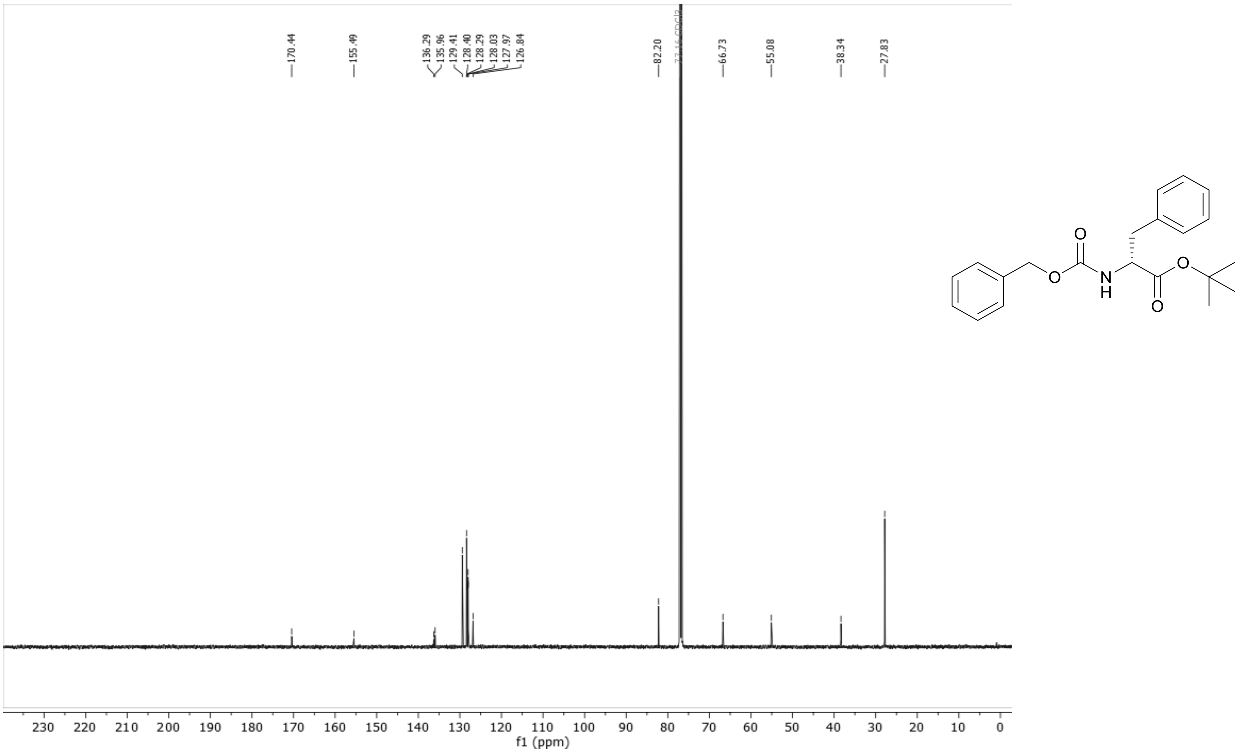

Figure S15. ^{13}C-NMR spectrum of synthesised Z-Phe-O^{t}Bu.

HPLC calibration curves

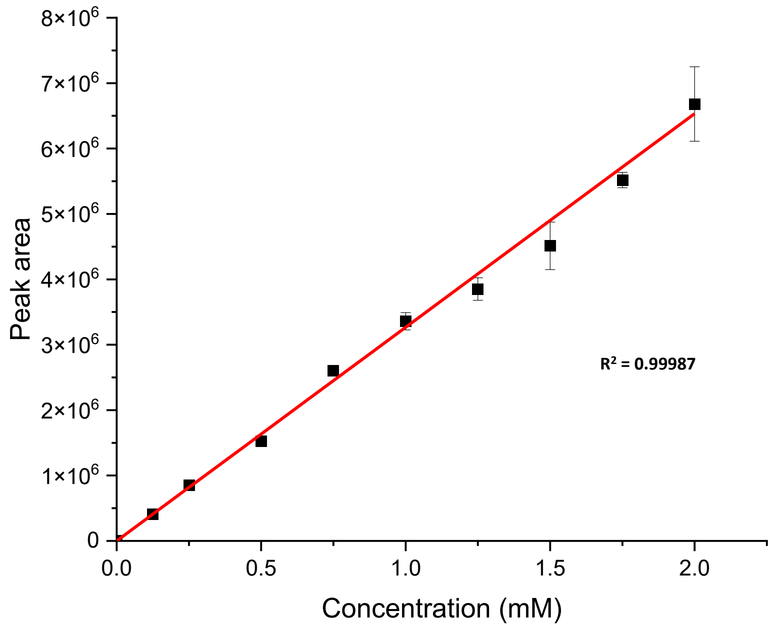

Figure S16. HPLC calibration curve for L-Phe (9.6 min) using HPLC method described on page 2.

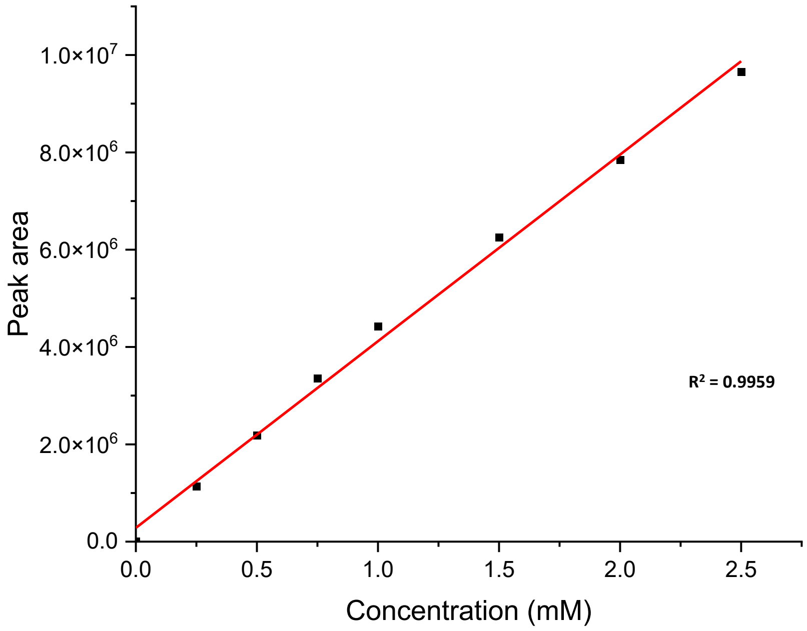

Figure S17. HPLC calibration curve for benzyl alcohol (14.2 min) using HPLC method described on page 2.

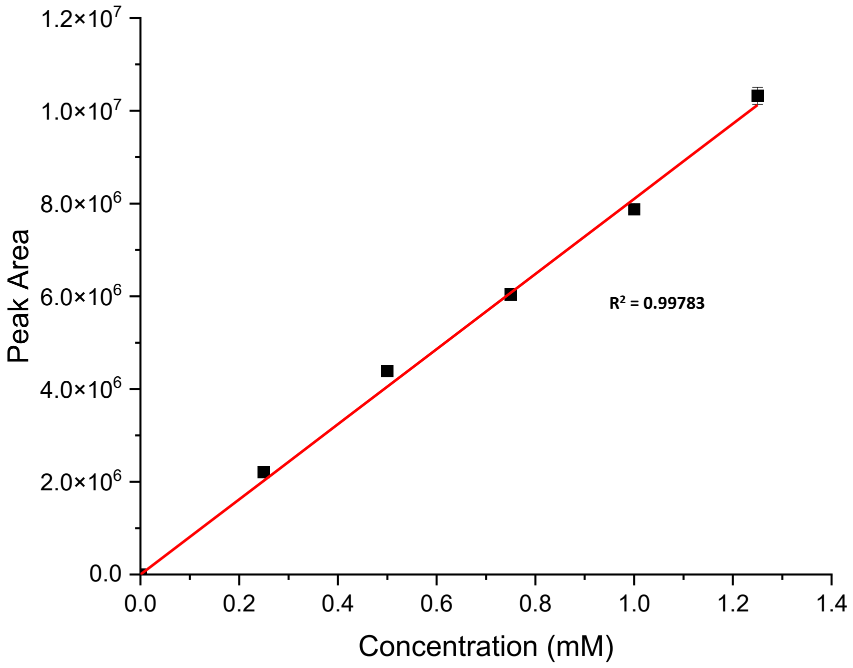

Figure S18. HPLC calibration curve for Z-L-Phe (20.1 min) using HPLC method described on page 2.

HPLC chromatograms

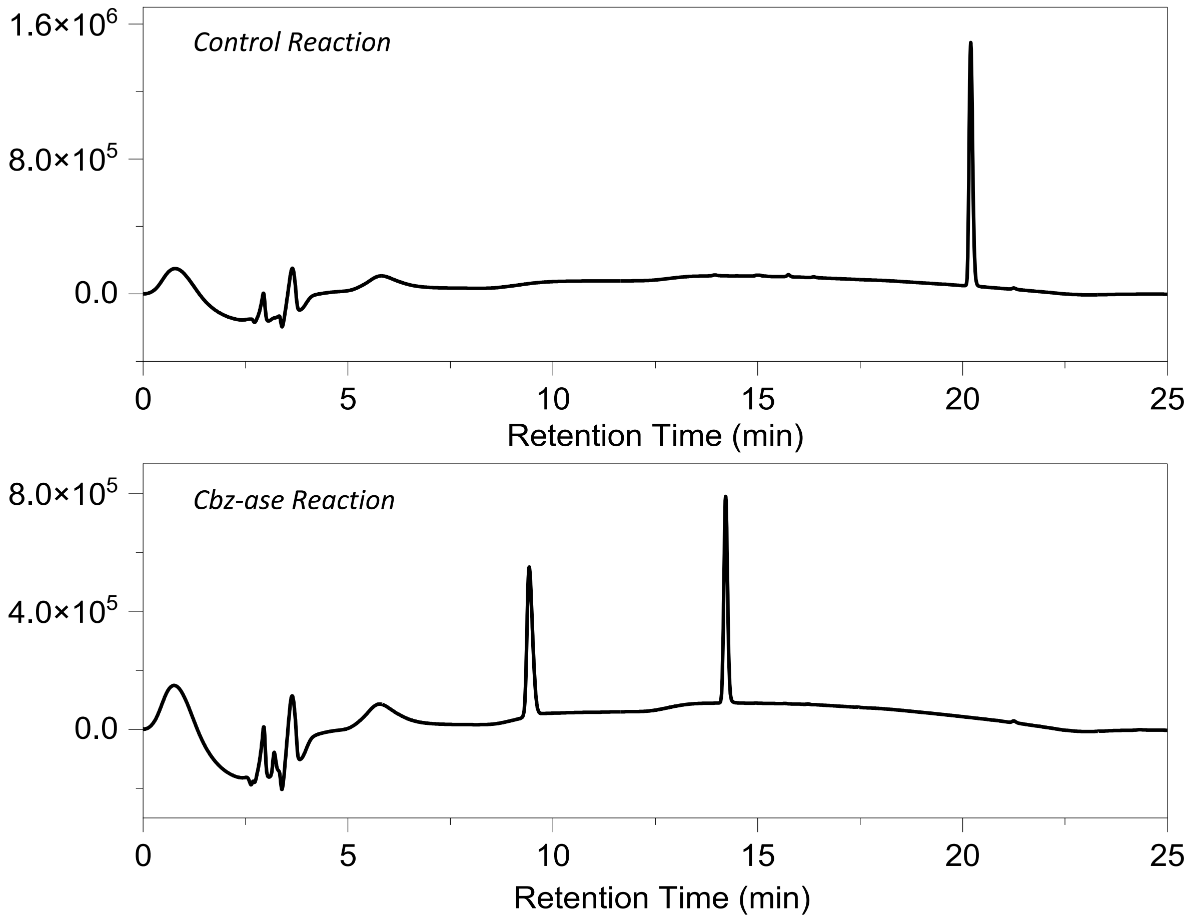

Figure S19. HPLC chromatograms for the deprotection of Z-L-Phe by Cbz-ase. Z-Phe = 20.1 min, benzyl alcohol = 14.2 min, L-Phe = 9.6 min.

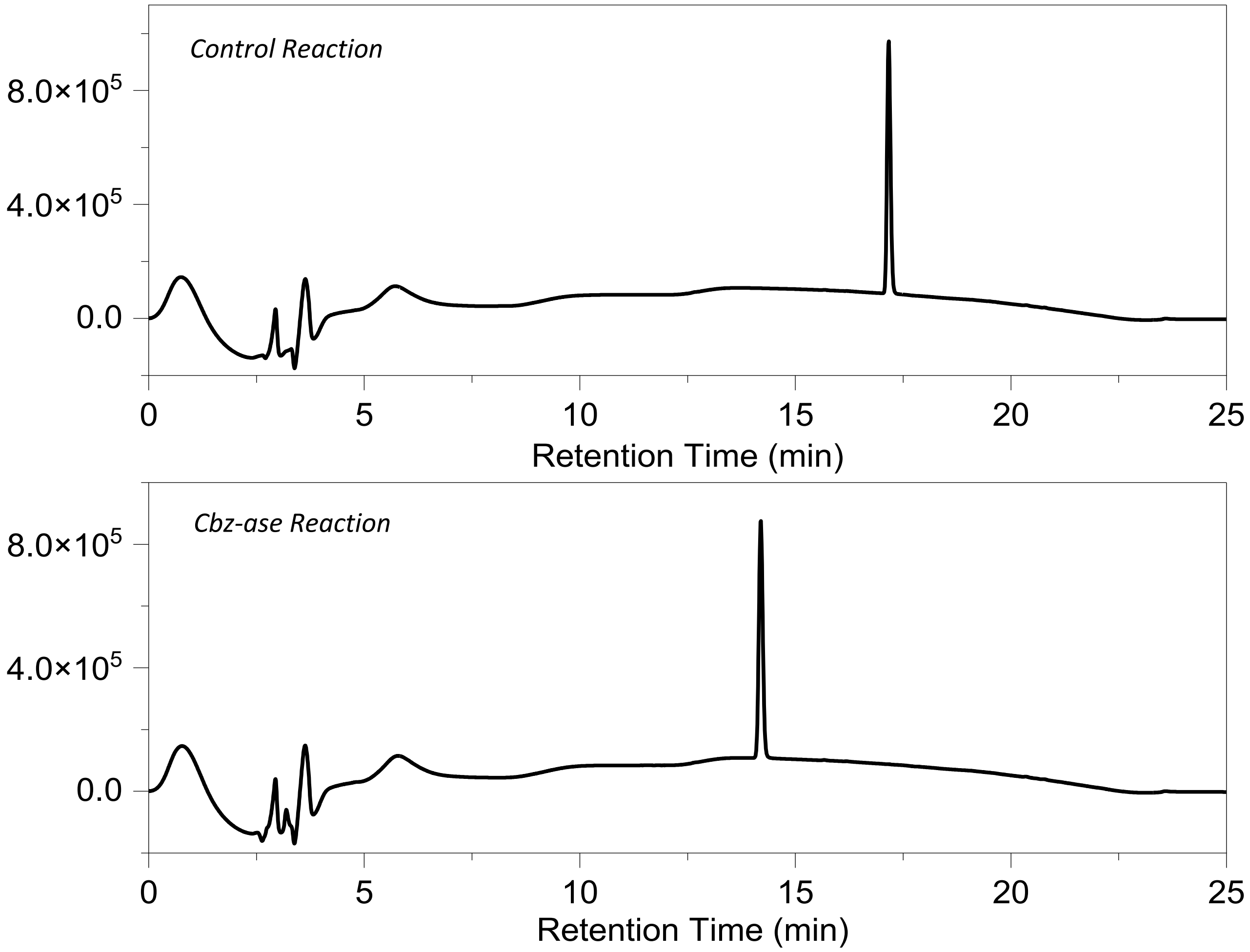

Figure S20. HPLC chromatograms for the deprotection of Z-L-Ala by Cbz-ase. Control reaction performed under same conditions but without the addition of Cbz-ase. Benzyl alcohol 14.2 min, Z-L-Ala = 17.2 min.

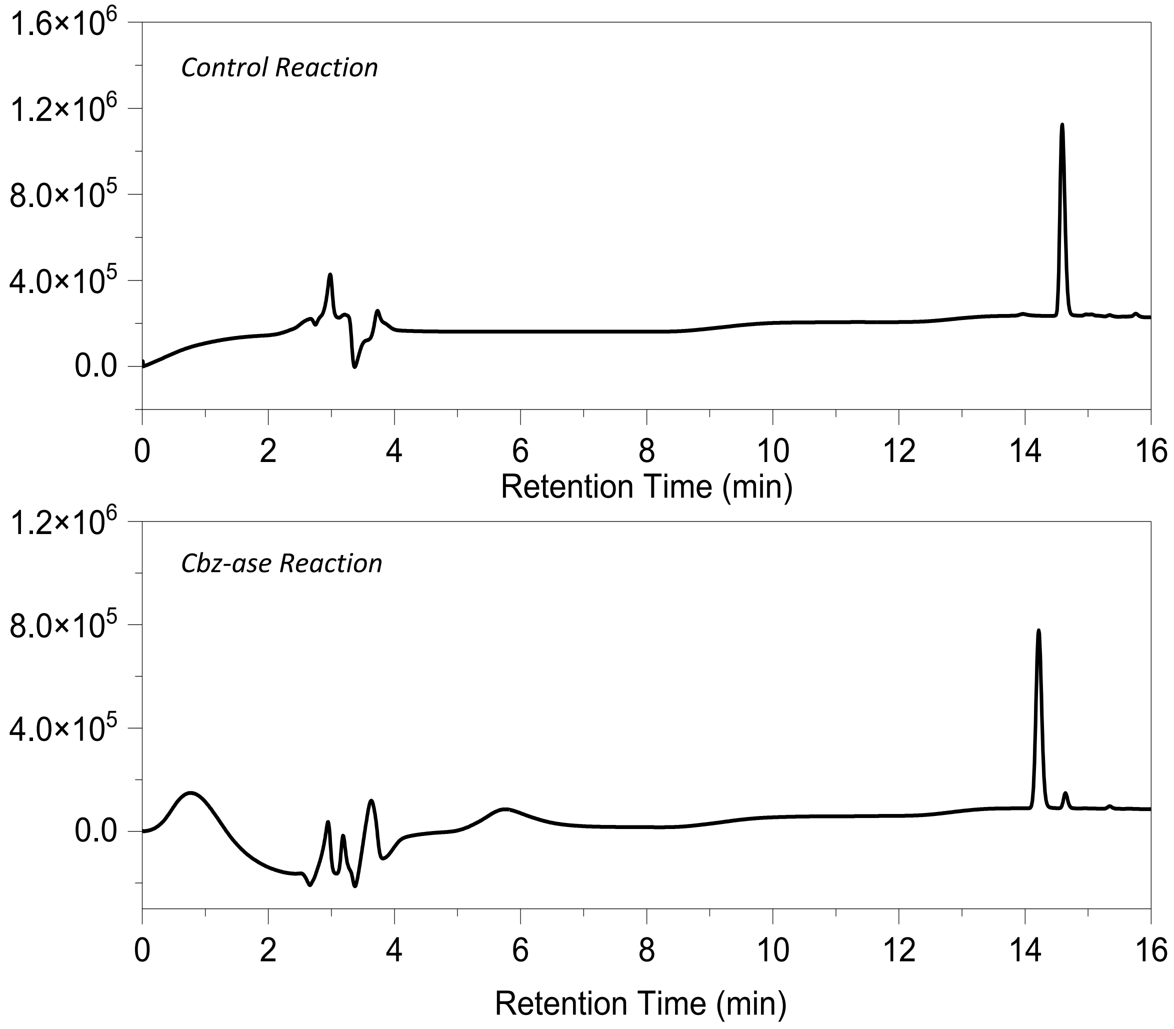

Figure S21. HPLC chromatograms for the deprotection of Z-Lys by Cbz-ase. Control reaction performed under same conditions but without the addition of Cbz-ase. Benzyl alcohol = 14.2 min, Z-Lys = 14.5 min.

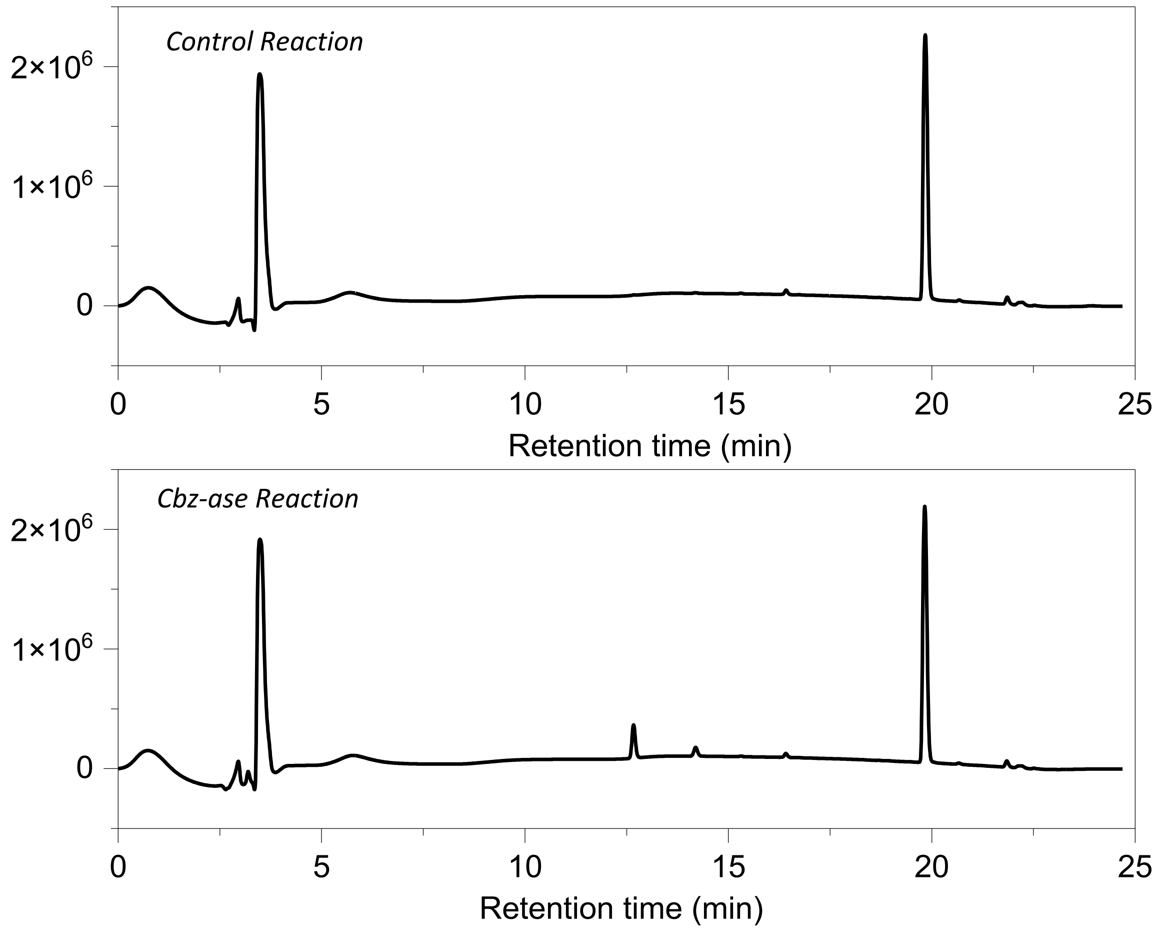

Figure S22. HPLC chromatograms for the deprotection of Z-Trp by Cbz-ase. L-Trp = 12.6 min, Benzyl alcohol = 14.2 min, Z-Trp = 19.9 min. Control reaction performed under same conditions but without the addition of Cbz-ase.

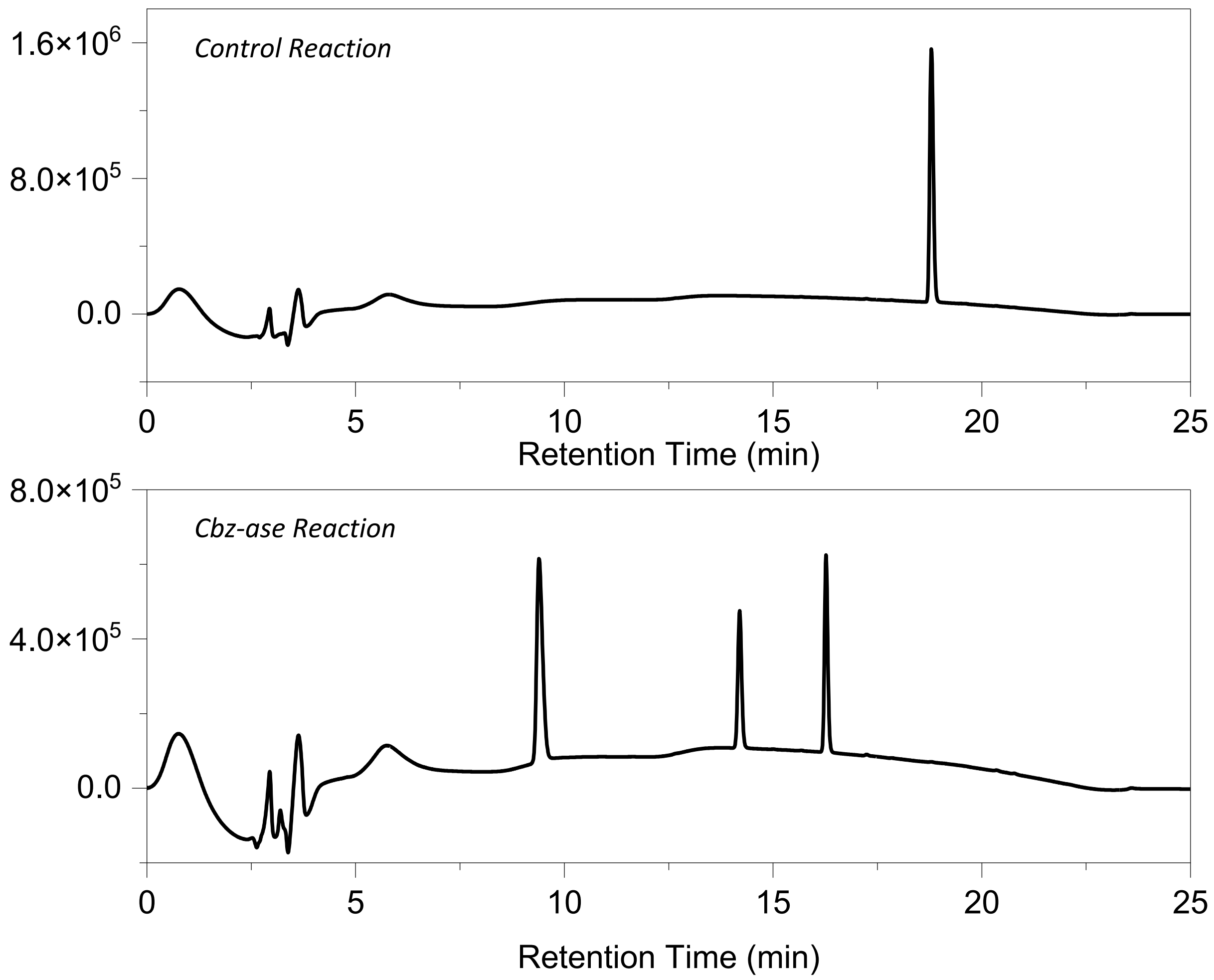

Figure S23. HPLC chromatograms for the deprotection of Z-Gly-Phe by Cbz-ase. L-Phe = 9.6 min, benzyl alcohol = 14.2 min, L-Gly-Phe = 16.3 min, Z-L-Gly-Phe = 18.9 min. Control reaction performed under same conditions but without the addition of Cbz-ase.

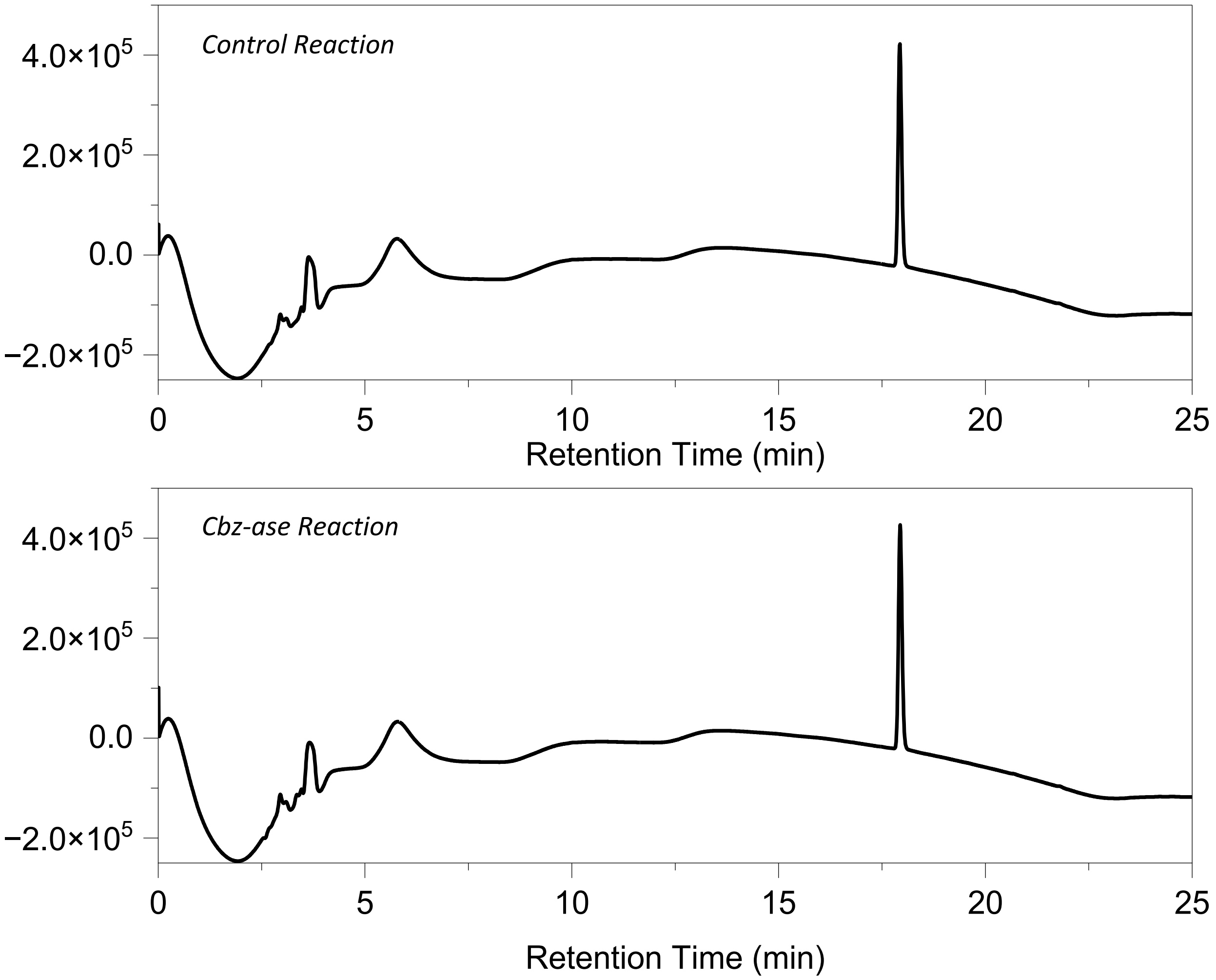

Figure S24. HPLC chromatograms for the deprotection of Z-Pro by Cbz-ase. Control reaction performed under same conditions but without the addition of Cbz-ase. Z-Pro = 18.0 min.

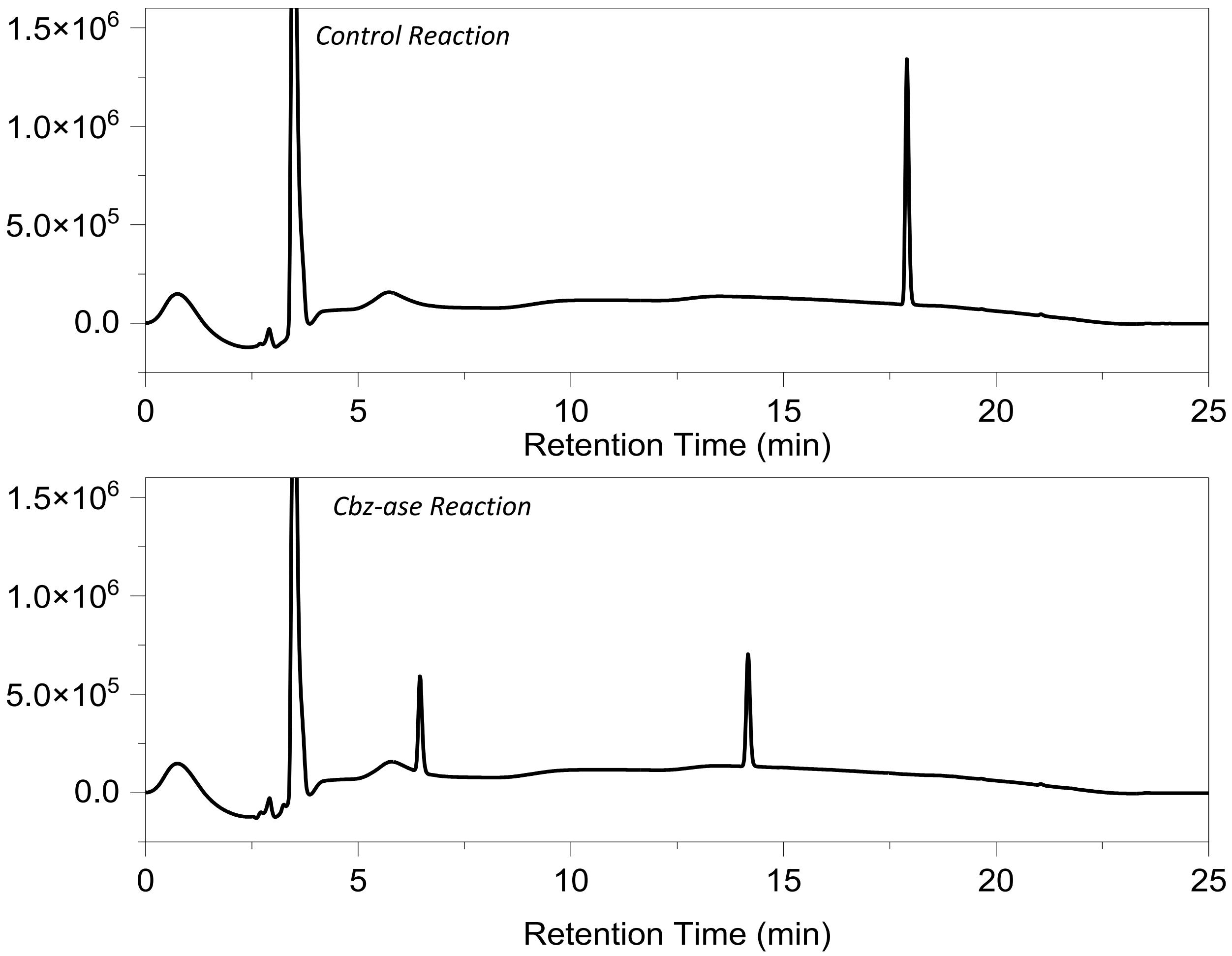

Figure S25. HPLC chromatograms for the deprotection of Z-Tyr by Cbz-ase. Control reaction performed under same conditions but without the addition of Cbz-ase. L-Tyr = 6.4 min, benzyl alcohol = 14.2 min, Z-Tyr = 18.0 min.

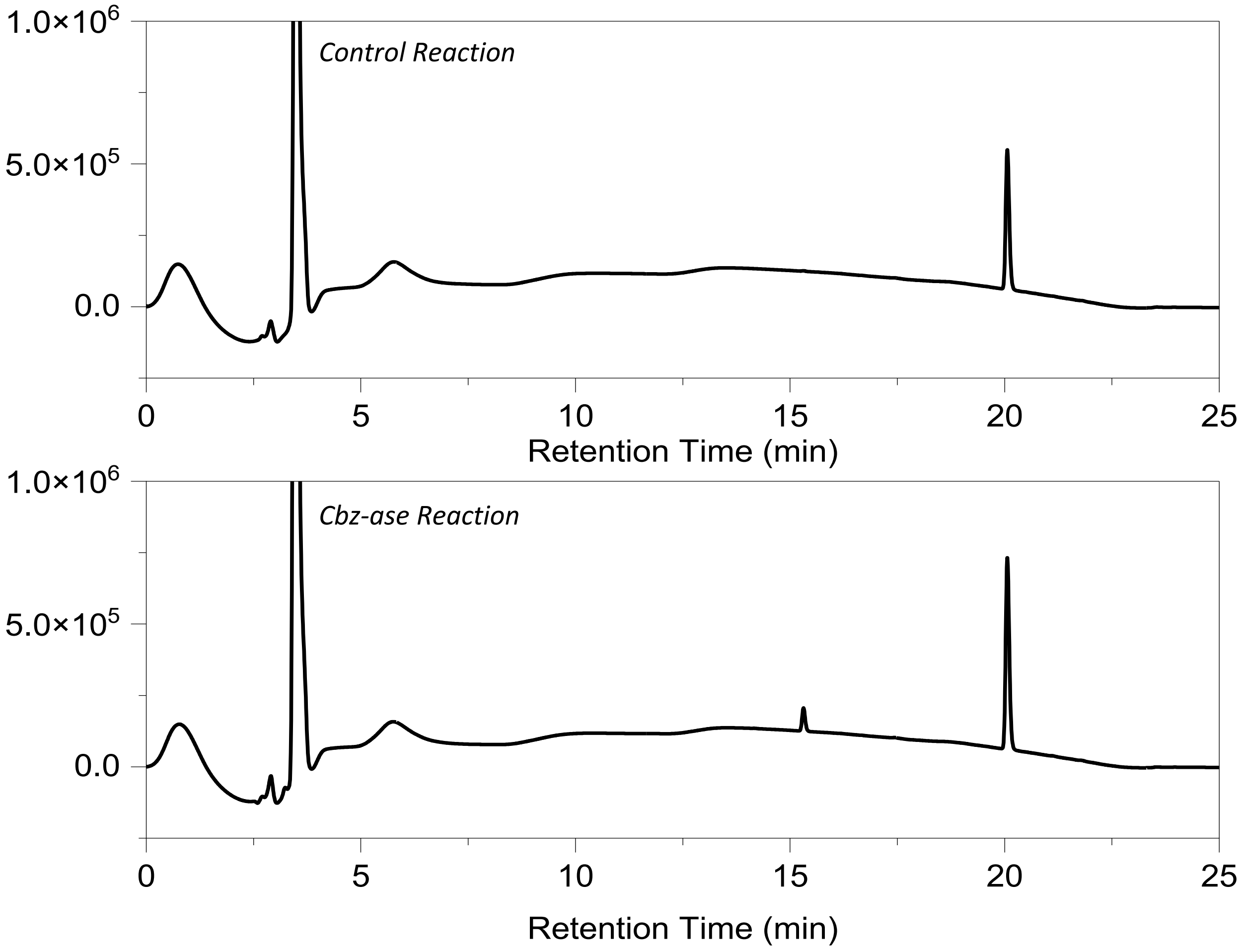

Figure S26. HPLC chromatograms for the deprotection of Boc-L-Lys(Z) by Cbz-ase. Control reaction performed under same conditions but without the addition of Cbz-ase. Boc-L-Lys(Z) = 20.2 min.

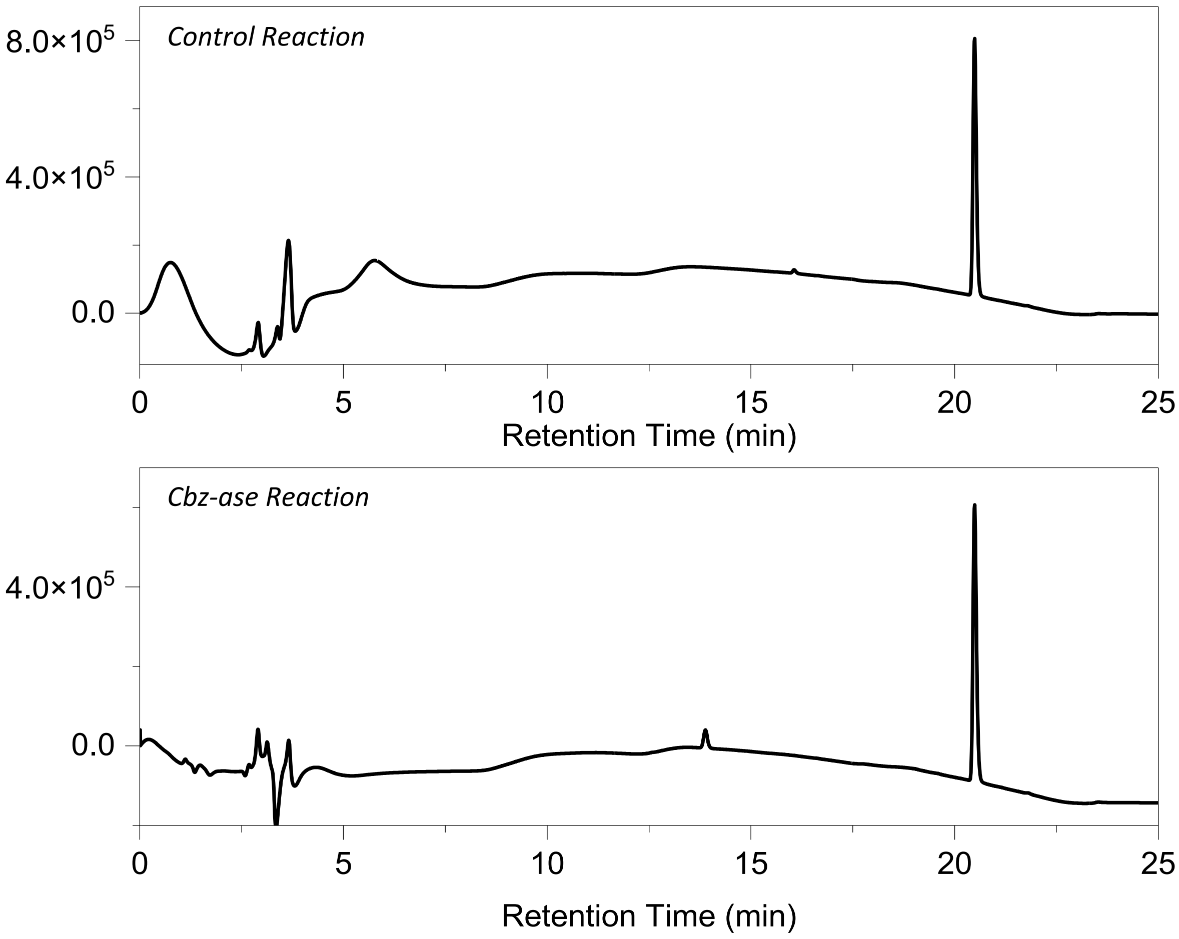

Figure S27. HPLC chromatograms for the deprotection of Z-Glu(O^{t}Bu) by Cbz-ase. Control reaction performed under same conditions but without the addition of Cbz-ase. Z-Glu(O^{t}Bu) = 20.5 min, benzyl alcohol = 14.2 min.

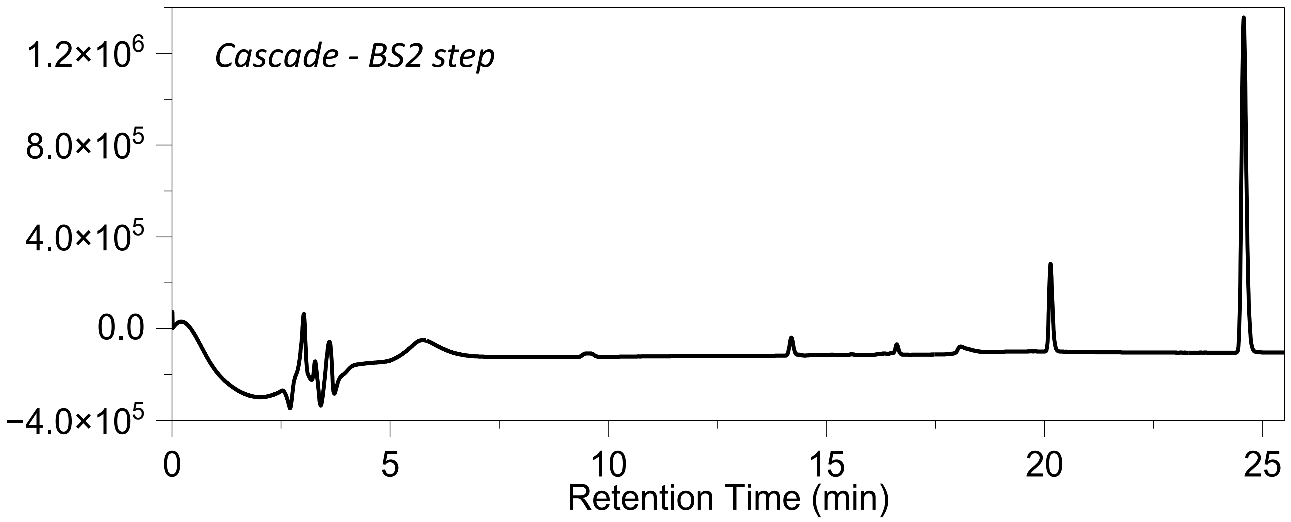

Figure S28. HPLC chromatogram for first step of the deprotection casecade in which BS2 removes the tert-butyl group from Z-Phe-O^{t}Bu (24.5 min). Z-Phe, product of this step = 20.1 min.

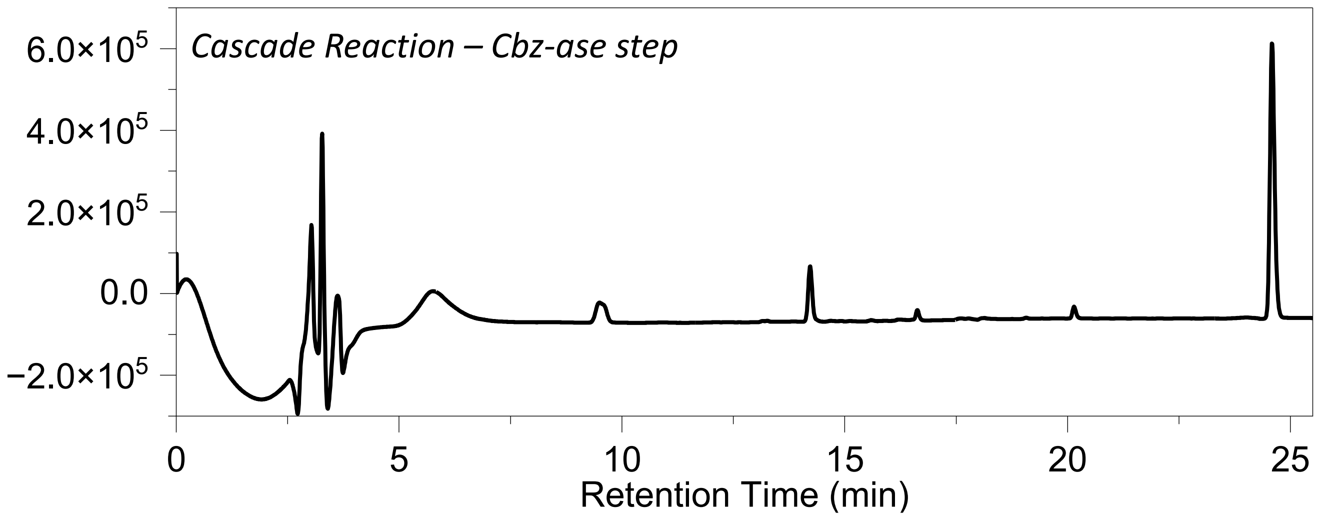

Figure S29. HPLC chromatogram for the second step of the deprotection cascade in which Cbz-ase hydrolyses the Cbz group of Z-Phe to yield the free L-Phe and benzyl alcohol. (Note: dilution occurs between steps due to addition of Cbz-ase). Retention times; L-Phe = 9.6 min, benzyl alcohol = 14.2 min, Z-Phe = 20.1 min, Z-Phe-O^{t}Bu = 24.4 min.

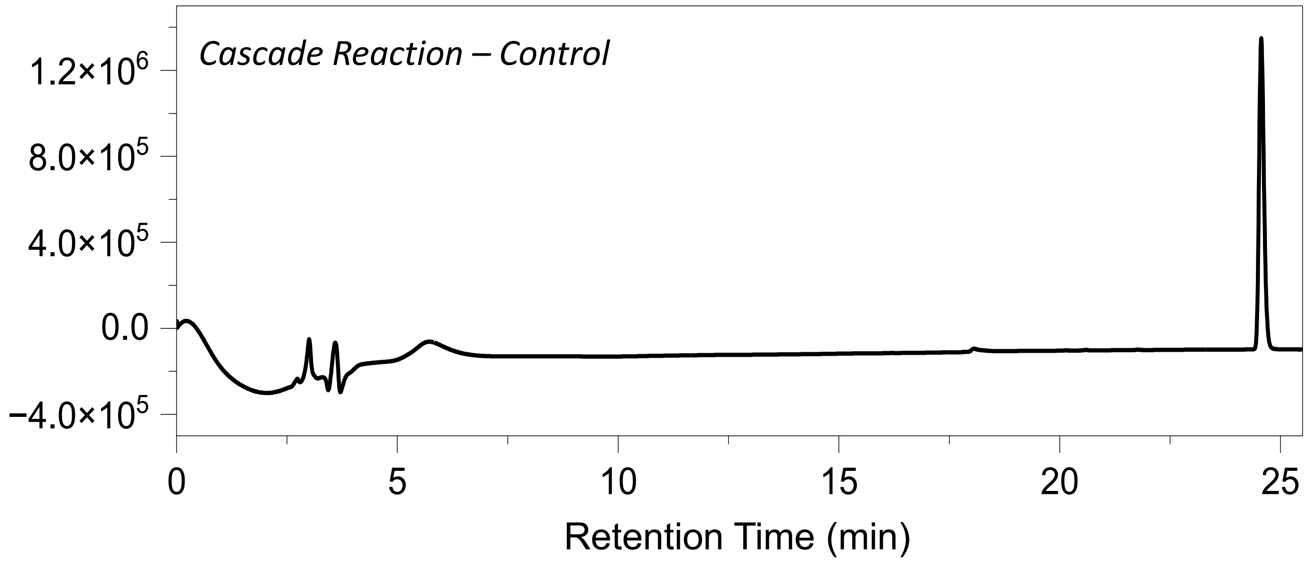

Figure S30. HPLC chromatogram for the control reaction for the deprotection cascade. Control was set-up in the same way as the enzyme reaction except no enzyme was added. No product was observed and only the substrate remains, Z-Phe-O^{t}Bu = 24.4 min.

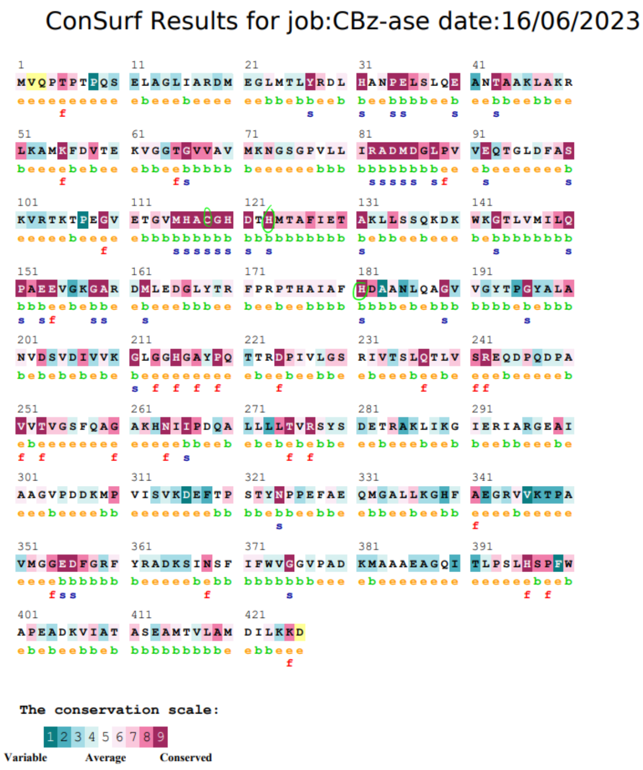

Figure S31. Evolutionary conservation analysis of Cbz-ase, showing conserved and variable residues.

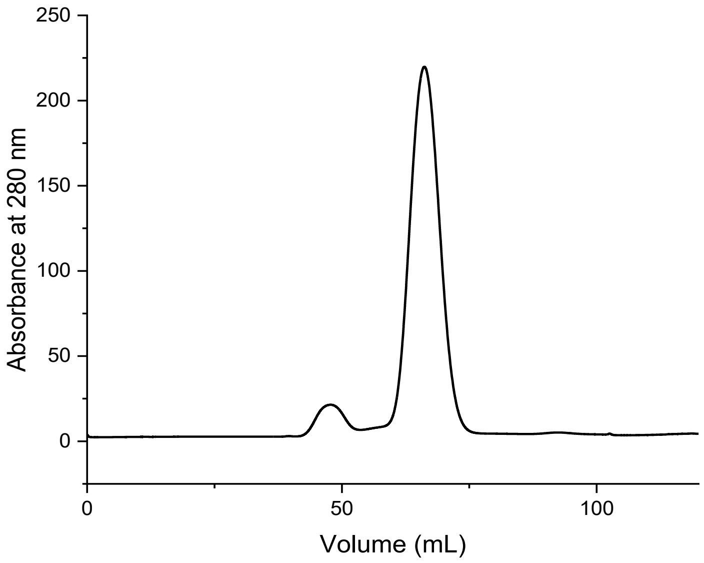

Figure S32. Size exclusion chromatogram for the Cbz-ase purification using a Superdex HiLoad 16/60 S200 column. Peak elution of 67 mL estimates the structure to be a tetrameric protein.

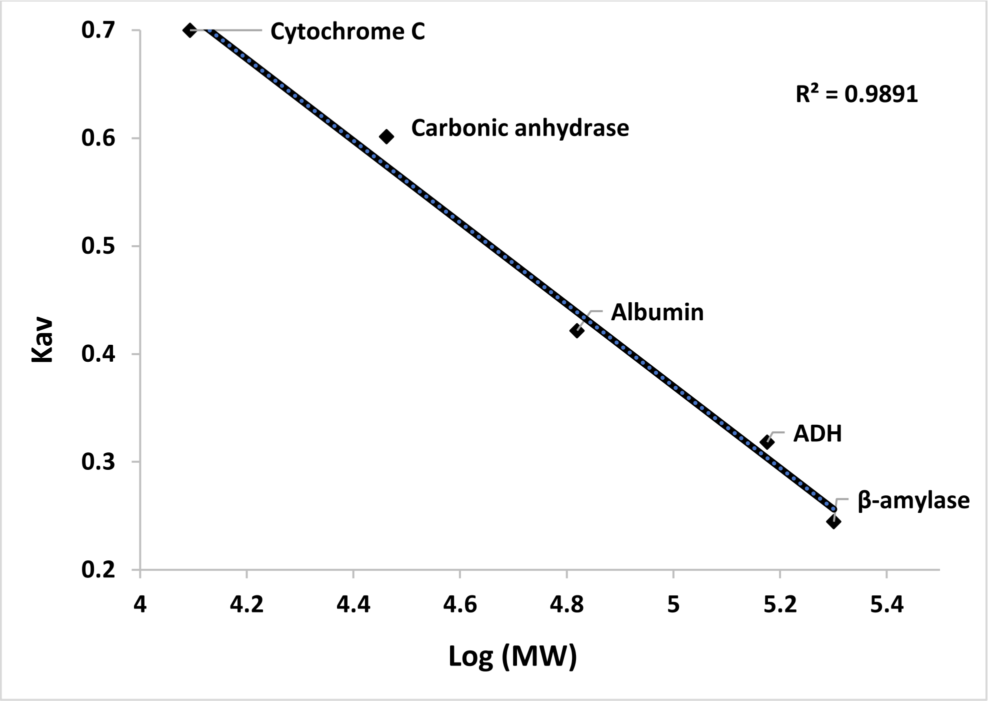

The molecular weight of each protein is estimated using the following equation:

$$MW = 10^{\frac{Kav-2.2653}{-0.379}}$$

$$Kav = \frac{Ve - Vo}{Vt - Vo}$$

Where:

MW is measured in Da

Kav = partition coefficient

Ve = Elution volume

Vo = Void volume (45.58 ml)

Vt = Total bed volume (120 ml)

Figure S33. Calibration curve of the Superdex HiLoad 16/60 S200 size exclusion chromatography column and corresponding equation used to estimate protein molecular weight (MW).

References

1. G. Sánchez-Carrón, T. Fleming, K. E. Holt-Tiffin and D. J. Campopiano, *Anal. Chem.*, 2015, **87**, 3923-3928.
2. U. T. Bornscheuer, G. R. Ordoñez, A. Hidalgo, A. Gollin, J. Lyon, T. S. Hitchman and D. P. Weiner, *J. Mol. Cat. B: Enzym.*, 2005, **36**, 8-13.
3. P. Strazzolini, M. Scuccato and A. G. Giumanini, *Tetrahedron.*, 2000, **56**, 3625-3633.
4. J.-H. Jian, H.-W. Zeng, T.-S. Kuo, P.-Y. Wu and H.-L. Wu, *Organic Letters*, 2019, **21**, 9468-9472.
5. J. Jumper, R. Evans, A. Pritzel, T. Green, M. Figurnov, O. Ronneberger, K. Tunyasuvunakool, R. Bates, A. Žídek, A. Potapenko, A. Bridgland, C. Meyer, S. A. A. Kohl, A. J. Ballard, A. Cowie, B. Romera-Paredes, S. Nikolov, R. Jain, J. Adler, T. Back, S. Petersen, D. Reiman, E. Clancy, M. Zielinski, M. Steinegger, M. Pacholska, T. Berghammer, S. Bodenstein, D. Silver, O. Vinyals, A. W. Senior, K. Kavukcuoglu, P. Kohli and D. Hassabis, *Nature*, 2021, **596**, 583-589.
6. M. Mirdita, K. Schütze, Y. Moriwaki, L. Heo, S. Ovchinnikov and M. Steinegger, *Nature Methods*, 2022, **19**, 679-682.
7. E. F. Pettersen, T. D. Goddard, C. C. Huang, E. C. Meng, G. S. Couch, T. I. Croll, J. H. Morris and T. E. Ferrin, *Prot. Sci.*, 2021, **30**, 70-82.
8. Y.-F. Lin, C.-W. Cheng, C.-S. Shih, J.-K. Hwang, C.-S. Yu and C.-H. Lu, *J. Chem. Inform. and Model.*, 2016, **56**, 2287-2291.
9. H. Ashkenazy, S. Abadi, E. Martz, O. Chay, I. Mayrose, T. Pupko and N. Ben-Tal, *Nucl. Acids Res.*, 2016, **44**, W344-W350.
10. J. Huang and A. D. Mackerell, *J. Comp. Chem.*, 2013, **34**, 2135-2145.
11. W. Tian, C. Chen, X. Lei, J. Zhao and J. Liang, 2018, *Nucl. Acids Res.*, **46**, W363-W367.
12. T. S. Girish, V. B, M. Colaco, S. Misquith and B. Gopal, *Acta Cryst. Sec. F Struct. Biol. & Crystall. Commun.*, 2013, **69**, 103-108.
